# An epigenetic screening determines BET proteins as targets to suppress self-renewal and tumorigenicity in canine mammary cancer cells

**DOI:** 10.1038/s41598-019-53915-7

**Published:** 2019-11-22

**Authors:** Pedro L. P. Xavier, Yonara G. Cordeiro, Pâmela A. Alexandre, Pedro R. L. Pires, Bruno H. Saranholi, Edson R. Silva, Susanne Müller, Heidge Fukumasu

**Affiliations:** 1 Laboratory of Comparative and Translational Oncology (LOCT), Department of Veterinary Medicine, Faculty of Animal Science and Food Engineering, University of Sao Paulo, Pirassununga, Brazil; 2 Present Address: CSIRO Agriculture and Food, Commonwealth Scientific and Industrial Research Organisation, Brisbane, Australia; 3 Department of Genetics and Evolution, Federal University of São Carlos, São Carlos, Brazil; 4 Department of Veterinary Medicine, Faculty of Animal Science and Food Engineering, University of Sao Paulo, Pirassununga, Brazil; 5 Structural Genomics Consortium, Buchmann Institute for Molecular Life Sciences, Johann Wolfgang Goethe University, Frankfurt am Main, Germany

**Keywords:** Breast cancer, Cancer stem cells

## Abstract

Targeting self-renewal and tumorigenicity has been proposed as a potential strategy against cancer stem cells (CSCs). Epigenetic proteins are key modulators of gene expression and cancer development contributing to regulation and maintenance of self-renewal and tumorigenicity. Here, we have screened a small-molecule epigenetic inhibitor library using 3D *in vitro* models in order to determine potential epigenetic targets associated with self-renewal and tumorigenicity in Canine Mammary Cancer (CMC) cells. We identified inhibition of BET proteins as a promising strategy to inhibit CMC colonies and tumorspheres formation. Low doses of (+)-JQ1 were able to downregulate important genes associated to self-renewal pathways such as WNT, NOTCH, Hedgehog, PI3K/AKT/mTOR, EGF receptor and FGF receptor in CMC tumorspheres. In addition, we observed downregulation of *ZEB2*, a transcription factor important for the maintenance of self-renewal in canine mammary cancer cells. Furthermore, low doses of (+)-JQ1 were not cytotoxic in CMC cells cultured in 2D *in vitro* models but induced G2/M cell cycle arrest accompanied by upregulation of G2/M checkpoint-associated genes including *BTG2* and *CCNG2*. Our work indicates the BET inhibition as a new strategy for canine mammary cancers by modulating the self-renewal phenotype in tumorigenic cells such as CSCs.

## Introduction

Mammary cancer in humans (HMC) and canines (CMC) share similar biological patterns, including high incidence, spontaneous development, associated risk factors, response to treatment and expression of molecular targets^1,2^. These features make dogs valuable models for comparative oncology and the development of new targets and therapies. Dogs have several advantages in comparison to other animal models such as generally sharing the same environment and being exposed to the same carcinogens as humans and thus influencing the epigenetic make-up^3^. Therefore, canine cancer cell lines are useful models to study tumor behavior and development, characterize and validate novel molecular targets and aid at the development of potential anticancer molecules for HMC.

Apart from tumorigenicity, intra-tumor heterogeneity, the presence of different tumor cells, including cancer-stem cells (CSCs), in a single tumor, greatly influence tumor development^4^. CSCs are rare cells within a tumor with the ability to self-renew, differentiate and tumor formation, underlying tumor initiation and progression^5^. Thus, considerable efforts to design innovative approaches to target these cells and their phenotypes have been made^6^. In order to enrich CSCs and, consequently, obtain a model to study and test approaches to target self-renewal and tumorigenicity, three-dimensional *in vitro* models (3D) using tumorspheres and colonies formation have been widely used^7^. However, in canine mammary cancer, few studies have addressed self-renewal and tumorigenicity phenotypes^8–10^. Recently, our group demonstrated that epithelial-mesenchymal transition (EMT)-associated transcription factors ZEB1 and ZEB2 are potential targets for the regulation of self-renewal and tumorigenicity of canine mammary cancer cells^11^. However, to the best of our knowledge, no chemical inhibitor for ZEB1/2 has thus far been developed^12^.

Although cancer is typically considered a genetic disease, epigenetic abnormalities play an important role in the development and progression of cancer^13^. Thus, inhibitors targeting epigenetic modulators (typically referred to as writers, erasers and readers) have recently gained interest as potential and innovative therapeutic approaches in cancer therapy^14,15^. In order to explore the therapeutic potential of novel epigenetic targets, specific inhibitors for a variety of epigenetic proteins have been developed. More than 50 specific inhibitors are available, covering particularly well the Bromodomain reader domains and epigenetic writers, histone lysine and arginine methyltransferases^16,17^.

The best-studied bromodomain family, is the bromodomain and extraterminal (BET) family of proteins. This family consists of four members: BRD2, BRD3, BRD4 and BRDT^18^. Each of these proteins possesses two bromodomains that read acetyl-lysine residues and influence gene regulation, such as recruitment a complex of regulatory proteins, including positive transcription elongation factor b (P-TEFb)^15,19,20^. BET proteins have been shown to play key roles in human cancer and are considered attractive therapeutic targets. Several small molecules inhibitors of BET proteins, including (+)-JQ1 and iBETs, exhibit anti-neoplastic effects in cancers, such as acute myeloid leukemia^21^, multiple myeloma^22^, NUT midline carcinoma^23^, colon cancer^24^ and breast cancer^25^. BET proteins are also associated with hypoxia and tumor angiogenesis^26^, epithelial-mesenchymal transition (EMT)^27^ and self-renewal^28^. On the other hand, in companion animals no clinical study has been performed this far apart from a study using dogs as models to test the toxicity of the BET inhibitor CPI-0610^29^.

Here, we use an approach to evaluate epigenetic targets in canine mammary cancer cells and show that BET inhibition by (+)-JQ1 is a promising strategy to inhibit self-renewal and tumorigenicity in CMC cells. Moreover, we demonstrate that BET proteins regulate the expression of genes associated with self-renewal and tumorigenicity pathways.

## Results

### Effect of epigenetic inhibitors on CMC cells

An initial screening was performed in order to determine the cytotoxic potential of a small library of 27 epigenetic inhibitors in the CF41.Mg cell line, considered the most malignant canine mammary cancer cell line of our cell bank, with higher tumorigenicity and self-renewal potential compared to the other cell lines^11^. From the 27 epigenetic inhibitors tested, only (+)-JQ1, NVS-CECR2-1 and UNC1999 showed an IC_50_ lower than 10 μM **(**Table 1**)**. According to the results, we set the non-cytotoxic concentration of 1 μM for all probes for the next experiments, which aim to observe the potential of the epigenetic inhibitors regarding tumorigenicity and self-renewal using 3D *in vitro* models.

**Table 1:** List of 27 epigenetic inhibitors, their targets and IC_50_ values.

Number	Inhibitor	Specific targets	Target Enzymatic Class	IC_50_ (µM)
1	(+)-JQ1	BRD2, BRD3, BRD4, BRDT(BET)	Bromodomains	3.9
2	GSK2801	BAZ2B/A	Bromodomains	>10
3	NI-57	BRPF	Bromodomains	>10
4	PFI-3	SMARCA2/4 e PB1/5	Bromodomains	>10
5	BAY-598	SET e SMYD2	Methyltransferases	>10
6	GSK-J4	JMJD3	Demethylases	>10
7	NVS-CECR2-1	CECR2-1	Bromodomains	3.98
8	GSK 484	PAD4	Deiminases	>10
9	SGC0946	DOT1L	Methyltransferases	>10
10	OICR-9429	WDR5	WD40	>10
11	R-PFI-2	SETD7	Methyltransferases	>10
12	MS049	PRMT4/6	Methyltransferases	>10
13	GSK864	IDH1	IDH1 mutant inhibitor	>10
14	GSK343	EZH2	Methyltransferases	>10
15	GSK-LSD1	LSD1	Demethylases	>10
16	MS023	PRMTs	Methyltransferases	>10
17	UNC1215	L3MBTL3	Methylated Lysines reader	>10
18	Bi-9564	BRD9/7	Bromodomains	>10
19	BAZ2-ICR	BAZ2A/B	Bromodomains	>10
20	UNC-1999	EZH2/1	Methyltransferases	4.70
21	TP-064	PRMT4	Methyltransferases	>10
22	A-196	SUV420H1/H2	Methyltransferases	>10
23	A-366	G9a/GLP	Methyltransferases	>10
24	PFI-4	BRPF1B	Bromodomains	>10
25	SGC-CBP30	CREBBP e EP300	Acetylases	>10
26	SGC-707	PRMT3	Methyltransferases	>10
27	GSK591	PRMT5	Methyltransferases	>10

### Assessment of epigenetic inhibitors on 3D *in vitro* models

**Figure 1:**
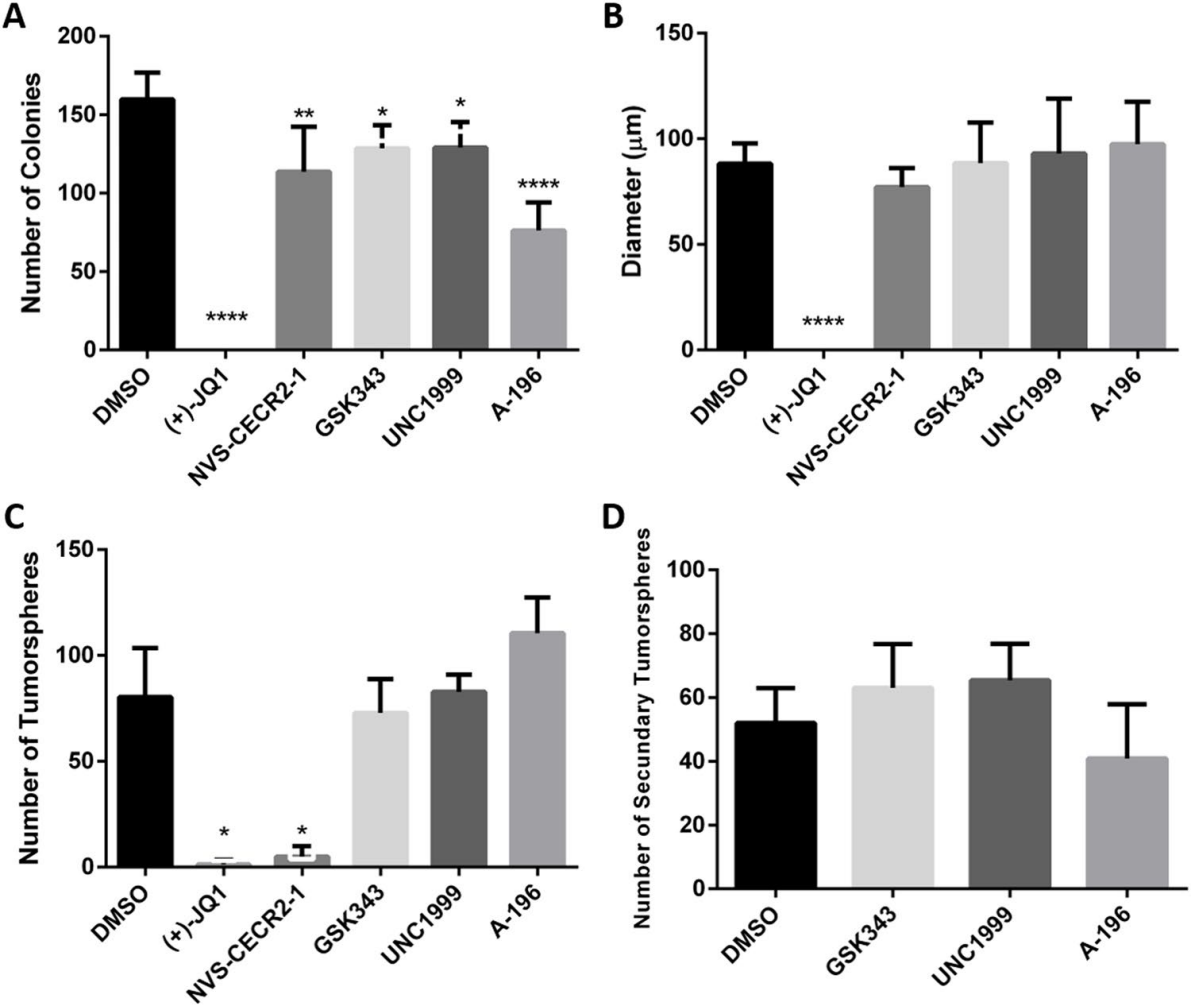
Number and size of colonies and number of primary and secondary tumorspheres. **(A)** (+)-JQ1, NVS-CECR2-1, GSK343, UNC1999 and A-196 decreased the number of colonies in comparison to the control (DMSO) formed in soft agar assay. (**B)** Only (+)-JQ1 decreased the diameter of colonies in comparison to the control (DMSO). Only ≥50 µm colonies were counted. (**C)** (+)-JQ1 and NVS-CECR2-1 inhibit tumorsphere formation in low-adherent plates. No difference was observed to tumorspheres treated with GSK343, UNC1999, and A-196 in comparison to the control (DMSO). (**D)** GSK343, UNC1999 and A-196 were also unable to inhibit the formation of secondary tumorspheres. (*p < 0.05; **p < 0.01; ****p < 0.0001; - One-way ANOVA followed by Tukey’s multiple comparison test).

Next, we aimed to explore the effects of epigenetic inhibitors regarding tumorigenicity and self-renewal of CF41.Mg cells using the tumor-cell colony formation in soft agar assay and the tumorsphere formation assay. From the 27 epigenetic inhibitors tested at 1 μM only (+)-JQ1, NVS-CECR2-1, GSK343, UNC1999 and A-196 decreased the number of colonies in soft agar when compared to the control treatment (Fig. 1A, P < 0.05) (Supplementary Fig. S1). However, only (+)-JQ1 was effective in reducing both the number and size of colonies in soft agar (Fig. 1B, P < 0.05). Therefore, these 5 epigenetic inhibitors were used in the assay for formation of primary and secondary tumorspheres, in which only (+)-JQ1 and NVS-CECR2-1 (at 1 μM) showed a significant inhibitory effect to primary tumorsphere formation (Fig. 1C; P < 0.05) (Supplementary Fig. S2). Both (+)-JQ1 and NVS-CECR2-1 nearly totally inhibited primary tumorspheres formation, while GSK343, UNC1999 and A-196 showed no inhibitory effect for primary and secondary tumorsphere formation **(**Fig. 1C,D) (Supplementary Fig. S3). Thus, (+)-JQ1 and NVS-CECR2-1 showed the most potent inhibitory effects in two 3D experiments and were selected for further investigation.

**Figure 2:**
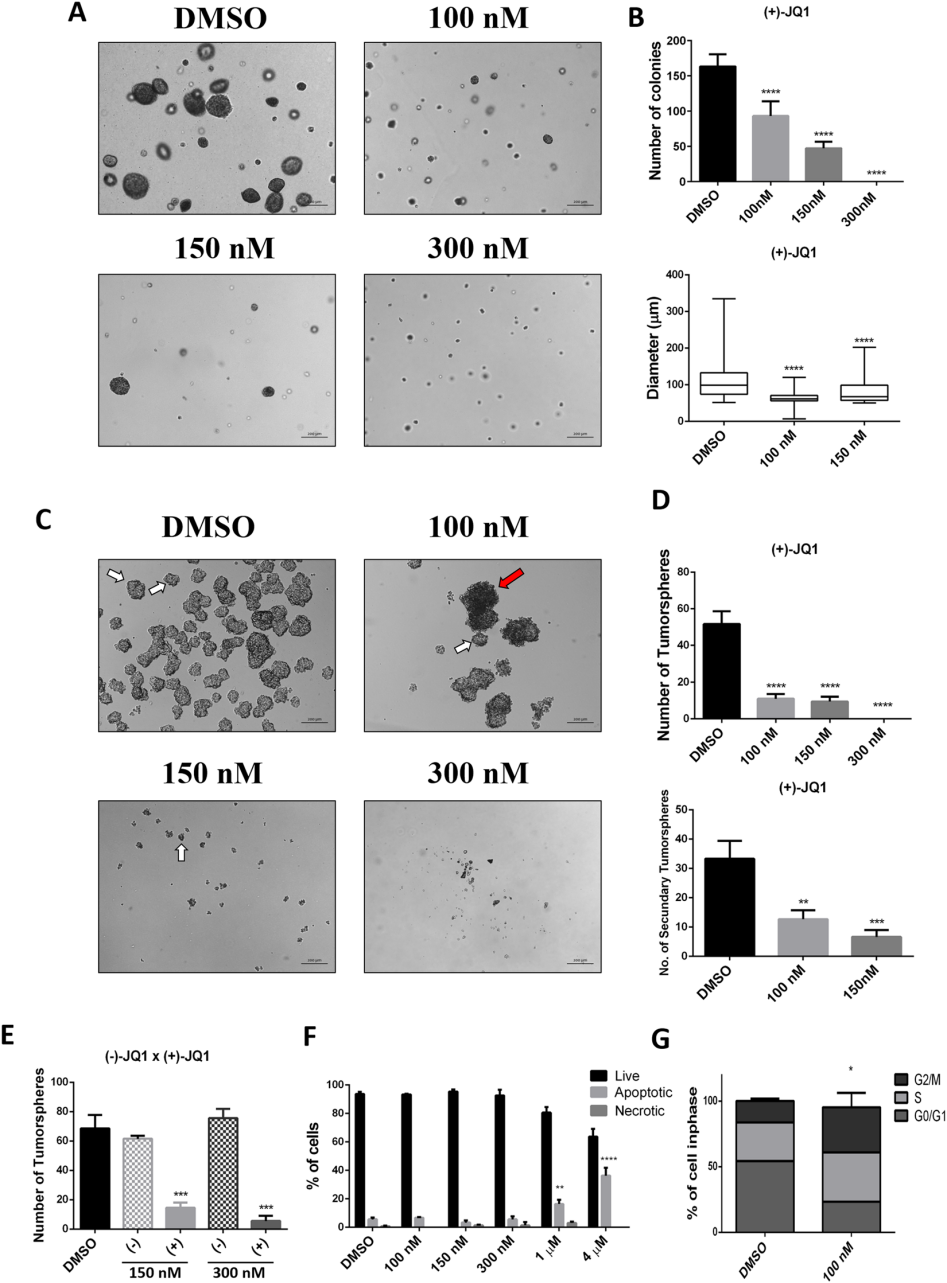
Effects of (+)-JQ1 regarding colonies formation, tumorsphere formation, cell death and cell cycle. **(A**,**B**) (+)-JQ1 used at concentrations of 100 nM, 150 nM and 300 nM were able to decrease the number and size of colonies in comparison to control. Only ≥50 µm colonies were counted. (**C**,**D)** In addition, (+)-JQ1 was able to inhibit the number of primary and secondary tumorspheres in comparison to the control. White arrows represent tumorspheres while red arrow represent cell aggregates. (**E**) The stereoisomer of (+)-JQ1, (−)-JQ1, at concentrations of 150 nM and 300 nM did not inhibit the tumorspheres formation. (**F**) One µM or 4 µM, respectively of (+)-JQ1 induced the increasing of apoptotic CF41.Mg cells. On the other hand, 300 nM, 150 nM and 100 nM of (+)-JQ1 showed no difference in comparison to the control (L = Live cells; A = Apoptotic cells). (**G)** After 72 h of (+)-JQ1 treatment, flow cytometry analyses for CF41.Mg cells show increase G2/M cell cycle arrest in (+)-JQ1 treated cells compared to the control. (**p < 0.01; ***p < 0.001; ****p < 0.0001 – One-way ANOVA followed by Tukey’s multiple comparison test).

We then evaluated the minimal concentration necessary to fully inhibit anchorage-independent cell growth. We tested (+)-JQ1 and NVS-CECR2-1 at lower concentrations in a dose dependent manner. (+)-JQ1 was able to fully inhibit the growth of colonies at the concentration of 300 nM, whereas at concentrations of 150 nM or 100 nM the number and size of the colonies was merely decreased (Fig. 2A,B; p < 0.0001). All (+)-JQ1 concentrations also inhibited the formation of tumorspheres (Fig. 2C,D; p < 0.0001) and concentrations of 150 nM or 100 nM of (+)-JQ1 reduced the number of secondary tumorspheres in comparison with the control (Fig. 2D; p < 0.0005). In order to confirm the specificity of the result, we next tested the inactive stereoisomer of (+)-JQ1, (−)-JQ1, at the same concentrations in CF41.Mg cells. This molecule has virtually the same physical and chemical structures as (+)-JQ1, but is unable to inhibit BET family bromodomains. Accordingly, there was no difference in the number of CF41.Mg tumorspheres between control and cells treated with (−)-JQ1, confirming that the effect on tumorsphere growth inhibition is due to inhibition of BET proteins (Fig. 2E).

Surprisingly, lower doses of NVS-CECR2-1 did not have the same effect on the growth of CF41.Mg tumorspheres (Supplementary Fig. S4). Upon closer inspection, we observed precipitates at concentrations above 1 μM, which can lead to cell death, justifying the initial result observed. Thus, we decided to concentrate on (+)-JQ1 in the further experiments.

Several reports show that at high concentrations (+)-JQ1 induces apoptosis and cell cycle arrest in human cancer cells^18,30,31^. Also, in CF41.Mg canine cells, concentrations of 1 µM and above of (+)-JQ1 induced apoptosis, whereas lower concentrations of 300 nM and below have no apoptotic effects (Fig. 2F; Supplementary Fig. S5A; P < 0.05). In order to explore the mechanism by which (+)-JQ1 inhibits colony and tumorspheres formation, we performed cell cycle analysis using flow cytometry. The cell cycle of CF41.Mg cells was analyzed using (+)-JQ1 at concentrations not inducting apoptosis. We established that (+)-JQ1 treatment induced a G2/M cell cycle arrest in these cells (Fig. 2G; Supplementary Fig. S5B; P < 0.05), suggesting a possible mechanism for the inhibition of the CF41.Mg tumorspheres and colonies.

We next confirmed the effect of (+)-JQ1 on tumorspheres in two other canine mammary cancer cell lines with tumorsphere potential, M5 and M25. (+)-JQ1 reduced the number of primary and secondary tumorspheres in M5 cells at both concentrations tested (150 nM and 300 nM) (Supplementary Fig. S6). In M25 cells, the number of secondary tumorspheres was reduced when treated with doses of 300 nM (+)-JQ1 (Supplementary Fig. S7).

### Transcriptomic analysis of (+)-JQ1-treated tumorspheres

**Figure 3:**
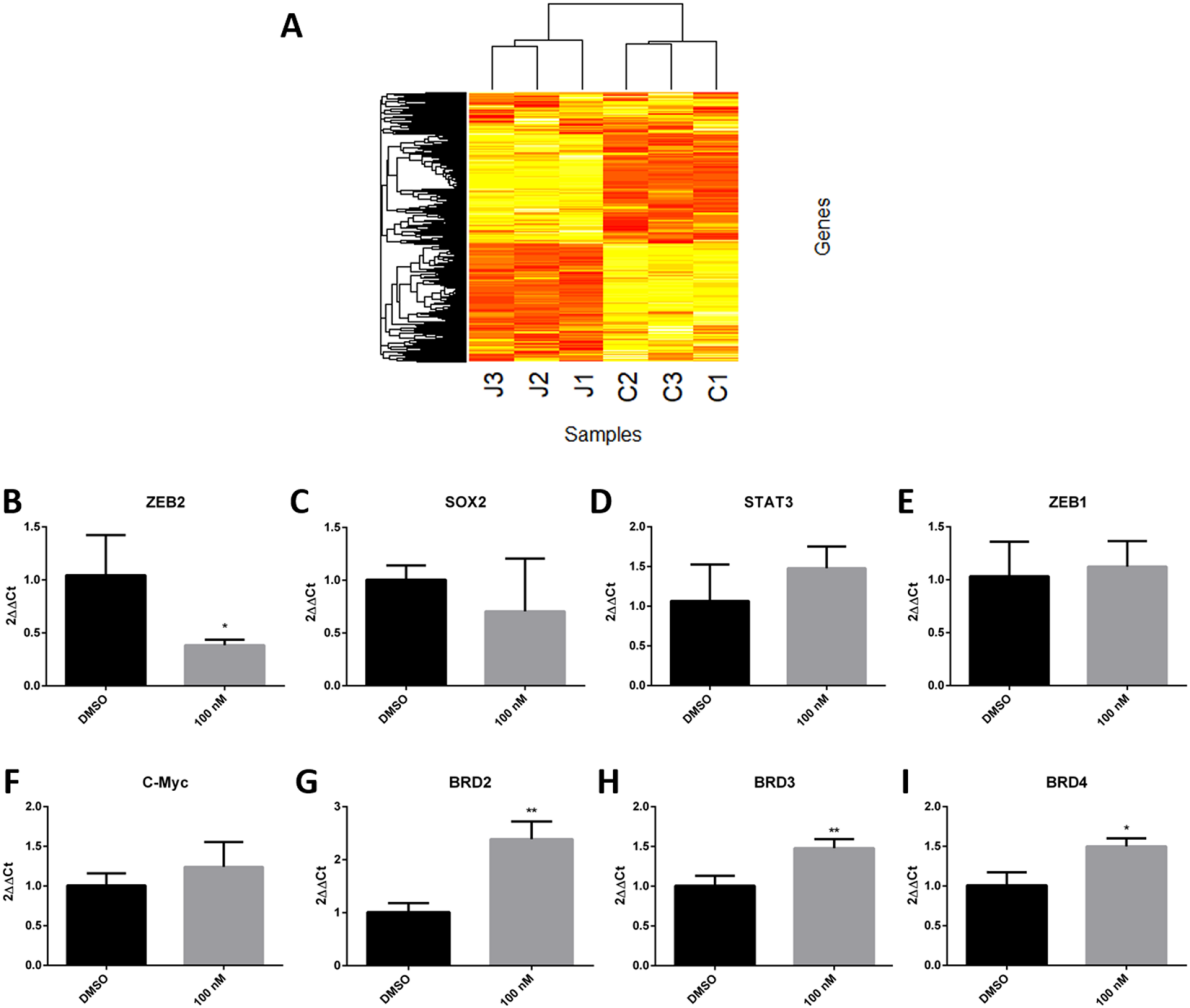
Gene expression analysis in (+)-JQ1-treated and non-treated CF41.Mg tumorspheres and adhrente cells. (**A**) Heatmap of all differentially expressed (DE) genes between (+)-JQ1-treated tumorspheres and (−)-JQ1-treated tumorspheres demonstrating the impact of (+)-JQ1 on modulation of gene expression in CF41.Mg tumorspheres. (**B**) (+)-JQ1 treatment significantly decreased *ZEB2* expression. (**C–E**) (+)-JQ1 treatment had no effect on gene expression of key stem cell associated genes, *SOX2*, *STAT3*, *ZEB1 and C-Myc*. (**F–I**) (+)-JQ1 treatment significantly increased the levels of *BRD2*, *BRD3* and *BRD4* gene expression. The 18* S* gene was used as the housekeeping gene. (*p < 0.05; **p < 0.01; - Unpaired T test).

In order to assess the genes affected by treatment with (++)-JQ1 in tumorspheres, we treated CF41.Mg tumorspheres with 100 nM of the inhibitor. An average of 19.8 million paired-end reads were sequenced per replicate (3 replicates per tumorspheres condition) and an average of 90% were aligned to the reference genome as concordant pairs (Supplementary Table S1). A total of 11,620 genes passed quality control and were tested for differential expression (DE). Of these 516 genes were downregulated and 444 were upregulated in (+)-JQ1-treated tumorspheres (FDR < 0.01 and LogFC > 1), demonstrating the impact of (+)-JQ1 in gene expression modulation on CF41.Mg tumorspheres even at a low dose (Fig. 3A). The top 25 up- and downregulated genes are exhibited in Table 2 and the full list is shown in the Supplementary Material (Supplementary Table S2). Interestingly, we found some of the top downregulated genes by (+)-JQ1 associated with self-renewal including Thrombospondin-2 (*THBS2*) (LogFC = −6.01), *ETV7* (LogFC = −4.31), Dickkopf-related protein 1 (*DKK1*) (LogFC = −4.12) and ROS proto-oncogene 1 (*ROS1*) (LogFC = −4.07)^32–35^. Functional enrichment analysis showed that DE genes between tumorspheres treated with (+)-JQ1 and (−)-JQ1 were related to KEGG and Reactome pathways such as proteoglycans in cancer, pathways in cancer, MicroRNAs in cancer, extracellular matrix organization, degradation of the extracellular matrix and regulation of insulin-like growth factor (IGF) transport and uptake by insulin-like growth factor binding proteins (IGFBPs). A full list of enriched terms is reported in Table 3.

**Table 2:** The top 25 down and upregulated genes in 100 nM (+)-JQ1-treated tumorspheres in comparison with control tumorspheres.

Ensembl ID	Gene name	logFC	FDR
ENSCAFG00000007251	*KRT5*	−7.18	1.81E-78
ENSCAFG00000015625	*FMN2*	−6.44	~0
ENSCAFG00000000874	*THBS2*	−6.01	3.46E-94
ENSCAFG00000018405	*FST*	−5.98	~0
ENSCAFG00000040526		−5.90	7.91E-61
ENSCAFG00000008359	*DCHS2*	−5.49	3.50E-154
ENSCAFG00000031666	*CLSTN3*	−5.43	1.92E-56
ENSCAFG00000006142	*DCN*	−5.30	~0
ENSCAFG00000024288	*TRABD2B*	−5.30	1.15E-107
ENSCAFG00000033166		−5.20	3.64E-33
ENSCAFG00000005068	*KCTD12*	−5.03	3.34E-21
ENSCAFG00000003684	*UCP1*	−5.01	3.97E-106
ENSCAFG00000006674	*SPINK5*	−5.00	1.68E-22
ENSCAFG00000017871	*FAT2*	−4.38	4.05E-39
ENSCAFG00000009666	*ZPLD1*	−4.38	6.80E-40
ENSCAFG00000005012	*TMEM163*	−4.38	6.24E-39
ENSCAFG00000001394	*ETV7*	−4.32	5.70E-20
ENSCAFG00000008335		−4.30	1.05E-24
ENSCAFG00000031918	*TMEM26*	−4.22	3.76E-40
ENSCAFG00000015553	*DKK1*	−4.12	6.80E-40
ENSCAFG00000000923	*ROS1*	−4.08	1.50E-52
ENSCAFG00000032756	*FAM198b*	−3.96	1.02E-127
ENSCAFG00000017137	*TENM2*	−3.95	4.58E-51
ENSCAFG00000006138	*LUM*	−3.93	~ 0
ENSCAFG00000015708	*ACTA2*	−3.93	2.33E-21
ENSCAFG00000009258	*CYTIP*	5.54	4.34E-21
ENSCAFG00000007018	*DEPP1*	5.27	3.18E-37
ENSCAFG00000014752	*TFP1*	4.64	1.88E-25
ENSCAFG00000013306	*APO4*	4.60	7.08E-24
ENSCAFG00000006413	*NCKAP1L*	4.31	1.39E-30
ENSCAFG00000001091	*TGFB1*	4.11	2.46E-49
ENSCAFG00000001106	*LAMA2*	4.10	7.64E-20
ENSCAFG00000009333	*CES1*	3.65	1.71E-77
ENSCAFG00000018146	*ALDH3A1*	3.54	4.63E-137
ENSCAFG00000017937	*CYP1A1*	3.39	7.32E-76
ENSCAFG00000018218	*AMOT*	3.30	1.25E-14
ENSCAFG00000009421	*MMP2*	3.19	2.76E-98
ENSCAFG00000001007	*FBXO32*	3.17	5.15E-119
ENSCAFG00000029721	*ZNF132*	3.15	8.90E-18
ENSCAFG00000029558	*BMF*	3.02	2.99E-86
ENSCAFG00000030120	*CSPG4*	2.96	1.57E-154
ENSCAFG00000010888	*UPK1B*	2.92	2.83E-70
ENSCAFG00000012396	*PHYHIPL*	2.91	7.23E-19
ENSCAFG00000031014	*FAM180A*	2.85	1.65E-18
ENSCAFG00000018047		2.82	6.75E-33
ENSCAFG00000011236	*AXIN2*	2.77	9.49E-32
ENSCAFG00000011002	*TINAGL1*	2.76	2.50E-256
ENSCAFG00000011595	*ARHGAP6*	2.75	2.07E-21
ENSCAFG00000006977	*ABTB2*	2.70	3.92E-82
ENSCAFG00000011913	*LAMB3*	2.64	5.13E-13

**Table 3:** KEGG and Reactome pathway analysis of DE genes between 100 nM JQ1-treated tumorspheres and control tumorspheres.

KEGG and Reactome pathways			
Pathway	Description	Count in gene set	FDR
cfa05418	Fluid shear stress and atherosclerosis	19 of 125	0.0022
cfa05205	Proteoglycans in cancer	24 of 183	0.0022
cfa05200	Pathways in cancer	45 of 475	0.0022
cfa05206	MicroRNAs in cancer	18 of 134	0.0070
cfa05146	Amoebiasis	13 of 86	0.0182
cfa04512	ECM-receptor interaction	12 of 76	0.0182
cfa04360	Axon Guidance	19 of 164	0.0182
cfa04933	AGE-RAGE signaling pathway	13 of 93	0.0221
cfa00340	Histidine Metabolism	6 of 22	0.0323
cfa04750	Inflammatory mediator regulation of TRP Channels	12 of 89	0.0373
cfa1474244	Extracellular matrix organization	31 of 253	0.0019
cfa422475	Axon guidance	30 of 275	0.0106
cfa381426	Regulation of Insulin-like Growth Factor (IGF) transport and uptake by Insulin-like Growth Factor Binding Proteins (IGFBPs)	15 of 107	0.0334
cfa2022090	Assembly of collagen fibrils and other multimeric structures	11 of 58	0.0334
cfa1630316	Glycosaminoglycan metabolism	16 of 111	0.0334
cfa162582	Signal Transduction	135 of 2177	0.0334
cfa1474290	Collagen formation	13 of 86	0.0334
cfa1442490	Collagen degradation	10 of 46	0.0334
cfa1266738	Developmental Biology	37 of 415	0.0334
cfa8874081	MET activates PTK2 signaling	7 of 26	0.0373
cfa8875878	MET promotes cell motility	8 of 36	0.0405

In our analysis, we also identified genes associated with G2/M cell cycle checkpoint and self-renewal when comparing the data set with information from publicly available data from the Molecular Signatures DataBase (MSigDB, http://www.broadinstitute.org/gsea/msigdb) and PANTHER classification system. We found important G2/M checkpoint-associated genes upregulated by (+)-JQ1 including B-cell Translocation Gene 2 (*BTG2*), Cyclin-G2 (*CCNG2*) and Epidermal Growth Factor Receptor (*EGFR*). (+)-JQ1 also increased the gene expression of Bone Morphogenetic Protein 7 (*BMP7*) and BCL-2-like protein 11 (*BCL2L11*), commonly called Bim, both associated with programmed cell death. Finally, we observed that (+)-JQ1 downregulated genes of important self-renewal pathways including WNT, NOTCH, Hedgehog, PI3K/AKT/mTOR, EGF receptor and FGF receptor, such as *GNB4*, *TLE2*, *BMPR1B*, *CDH10*, *ACVR1B*, *ACTA2*, *FAT2*, *WNT9A*, *CDH13*, *PRKD1*, *PIK3CD*, *ERBB3*, *NRG1*, *GNB4*, *GNB5*, *NOS3*, *EIF4E3*, *PTPN6*, *PTCH1*, *BMP4* and *NOTCH1* (Table 4). Furthermore, we observed downregulation of *ZEB2*, a transcription factor important for the maintenance of self-renewal in canine mammary cancer cells expression^11^. The decrease of *ZEB2* expression in (+)-JQ1-treated CF41.Mg cells was also observed by qPCR analysis (Fig. 3B; P < 0.05). However, the expression levels of some self-renewal associated genes such as *SOX2*, *STAT3*, *ZEB1* and *C-Myc*, a key target gene of BET proteins, showed no significant difference after (+)-JQ1 treatment (Fig. 3C–F). Interestingly, (+)-JQ1 treatment significantly increased gene expression of *BRD2*, *BRD3* and *BRD4* in CF41.Mg cells (Fig. 3G–I; P < 0.05), which may present a compensatory response after BET proteins inhibition by the probe.

**Table 4:** Self-renewal-associated genes downregulated by (+)-JQ1.

Self-renewal-associated genes			
Pathway	Gene	LogFC	FDR
WNT	*GNB4*	−1.16	2.24E-30
	*TLE2*	−1.85	4.98E-24
	*BMPR1B*	−1.12	4.27E-11
	*CDH10*	−2.96	6.20E-15
	*ACVR1B*	−1.06	2.07E-18
	*ACTA2*	−3.93	2.33E-21
	*FAT2*	−4.38	4.05E-39
	*WNT9A*	−1.51	1.43E-21
	*CDH13*	−3.85	9.99E-19
EGFR	*PRKD1*	−2.16	6.72E-11
	*PIK3CD*	−1.49	5.05E-60
	*ERBB3*	−2.28	9.11E-15
	*NRG1*	−1.05	1.85E-67
PI3K/AKT/mTOR	*GNB4* *GNB5* *NOS3* *EIF4E3*	−1.16 −1.08 −2.59 −2.38	2.24E-30 5.11E-14 2.73E-16 7.08E-09
FGFR	*PTPN6*	−2.06	1.08E-26
	*PIK3CD*	−1.49	5.05E-60
Hedgehog	*PTCH1* *BMP4*	−1.07 −1.28	4.86E-26 1.03E-84
NOTCH	*NOTCH1*	−1.00	8.03E-77

These results demonstrate that BET inhibition by (+)-JQ1 can modulate key genes associated with self-renewal and G2/M checkpoint in CMC cells corroborating the decrease of tumorspheres and colonies accessed by 3D *in vitro* models.

### Canine BET proteins: gene expression and homology

**Figure 4:**
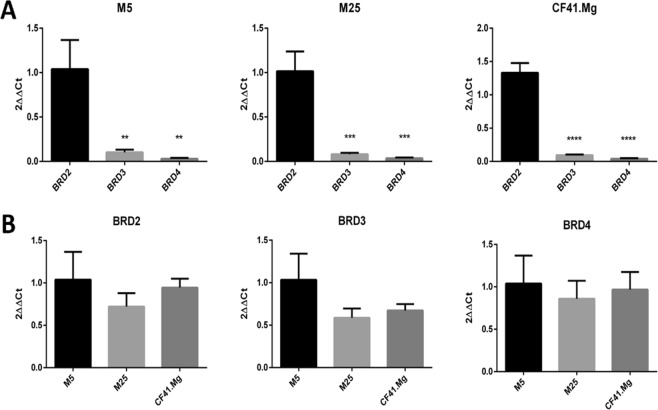
Gene expression analysis of BRD2, BRD3 and BRD4 in CMC cells. **(A)** The *BRD2* gene showed higher expression than the *BRD3* and *BRD4* genes in the M5, M25 and CF41 cell lines. (**B)** There was no difference in expression of the BRD2, BRD3 and BRD4 genes between M5, M25 and CF41.Mg cells. The *18 S* gene was used as the housekeeping gene. (*p < 0.05; **p < 0.01; ***p < 0.001; ****p < 0.0001 – One-way ANOVA followed by Tukey’s multiple comparison test).

BET proteins are extremely conserved between species and also their expression patterns has been found to be conserved^36^. In order to confirm the expression of *BRD2*, *BRD3* and *BRD4* in CMC cells we performed qPCR analysis. *BRD2*, *BRD3* and *BRD4* genes were expressed in CMC cells with *BRD2* being the most expressed gene (Fig. 4A). All three cell lines, M5, M25 and CF41.Mg, showed high expression of *BRD2*, *BRD3* and *BRD4* (Table 5) with no difference in expression level between the cell lines (Fig. 4B).

**Table 5:** Cycle Threshold (CT) values to *18S*, *BRD2*, *BRD3* and *BRD4* expression of M5, and M25 and CF41.Mg cells.

Samples	CT *18*S	CT *BRD2*	2ΔΔCT *BRD2*	CT *BRD3*	2ΔΔCT *BRD3*	CT *BRD4*	2ΔΔCT *BRD4*
M5	13.24 ± 0.48	21.67 ± 0.10	1.03 ± 0.32	25 ± 0.15	1.03 ± 0.30	26.75 ± 0.09	1.03 ± 0.33
M25	13.10 ± 0.40	22.02 ± 0.14	0.72 ± 0.15	25.65 ± 0.25	0.58 ± 0.10	26.86 ± 0.10	0.86 ± 0.21
CF41.Mg	13.17 ± 0.34	21.69 ± 0.23	0.94 ± 0.10	25.52 ± 0.44	0.67 ± 0.07	26.76 ± 0.17	0.96 ± 0.20

**Figure 5:**
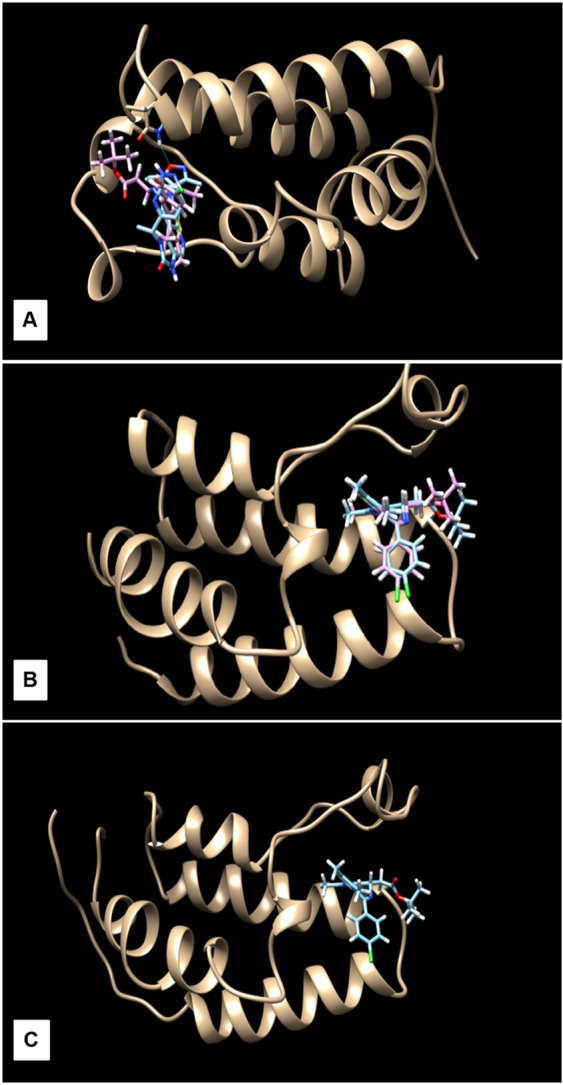
Docking experiments using the structure of the canine BRD2 **(A)**, BRD3 **(B)** and BRD4 **(C)** proteins with the (+)-JQ1 ligand. The BET inhibitor (+)-JQ1 was able to bind to all canine BET proteins.

The inhibitor (+)-JQ1 was designed based on the acetylated lysine binding sites of human BET proteins^18^. Thus, we performed *in silico* analysis to observe if (+)-JQ1 would be predicted to inhibit canine BET proteins. First, a comparative analysis between the amino acid sequences of human (BETh) and canine (BETc) proteins and the homology of the two bromodomains of the human and canine BET proteins, respectively was performed. Each of the BET protein members evaluated, was highly conserved between human and dog, with amino acid identity ranging from 94–100% (Table 6), suggesting that (+)-JQ1 is able to bind to the acetylysine binding site of canine BET proteins and displace them from chromatin. Finally, an *in silico* docking study between a (+)-JQ1 molecule and the canine BRD2, BRD3 and BRD4 proteins corroborated the binding of the inhibitor to canine BET proteins (Fig. 5).

**Table 6:** Evaluation of homology between human and canine BET proteins.

BETh	BETc	Query Cover	Identity
BRD2h	BRD2c	100%	98%
BRD2h (74–180)	BRD2c (74–180)	100%	100%
BRD2h (349–450)	BRD2c (349–450)	100%	100%
BRD3h	BRD3c	95%	94%
BRD3h (30–140)	BRD3c (30–140)	100%	99%
BRD3h (311–412)	BRD3c (311–412)	100%	95%
BRD4h	BRD4c	90%	97%
BRD4h (58–164)	BRD4c (58–164)	100%	99%
BRD4h (353–454)	BRD4c (353–454)	100%	100%

## Discussion

In the present study, we report the screening of a small-molecule epigenetic inhibitors (probes) library to modulate tumorigenicity and self-renewal phenotypes of canine mammary cancer cells. From 27 probes targeting different classes of epigenetic proteins we demonstrated that inhibition of BET proteins by (+)-JQ1 reduced the number of canine mammary colonies and tumorspheres already at concentrations of 100 nM. At these low doses (+)-JQ1 did not induce apoptosis in CF41.Mg canine cells, whereas at concentration of 1 µM and above apoptotic effects were observed (Fig. 2F,G; P < 0.05). This was accompanied by G2/M cell cycle arrest as opposed to apoptosis observed at higher concentrations. Furthermore, BET inhibition altered the expression of genes associated with self-renewal pathways including WNT, NOTCH, Hedgehog, PI3K/AKT/mTOR, EGFR and FGFR. Finally, low concentrations of (+)-JQ1 showed no cytotoxicity in CMC cells cultured in 2D *in vitro* models, suggesting BET inhibition as promising strategy to target tumorigenicity and self-renewal in CMC cells.

In this study, we showed that (+)-JQ1 targets anchorage-independent cells within canine mammary cancer cell populations. (+)-JQ1 treatment decreased colonies and tumorspheres formation by ~2-fold and ~6-fold, respectively at treatment concentrations of 100 nM. In contrast, high concentrations of (+)-JQ1 (~4 µM) reduced CF41.Mg cell numbers by 50% of when cultured in 2D *in vitro* model. Compounds that preferentially target CSCs in human breast cancer cells populations have been described previously. Two main studies have demonstrated the effects of salinomycin and metformin, substances well-known for antibacterial and antidiabetic properties, in breast cancer CSCs^37,38^. However, so far, only a few studies have demonstrated the effects of (+)-JQ1 specifically in CSCs phenotypes^28,39^ and, to our knowledge, this is the first study to demonstrate these effects in canine mammary cancer cells.

The inhibitor (+)-JQ1 inhibits specifically the family of epigenetic readers known as BET proteins (BRD2, BRD3, BRD4 and BRDT)^18^. BRD4 is a key mediator of MYC driven transcriptional programs in c-MYC driven tumors^40^. In human breast cancer, BRD4 plays an important role for breast tumor proliferation^41^ and BET inhibition has been shown to contribute to overcoming resistance in HER2 and hormone receptors positive tumors (HR)^42,43^. However, triple-negative breast cancer (TNBC), the most aggressive subtype, is not commonly associated with BRD4/MYC regulation^44,45^. These results suggest that BRD4/MYC is not the sole mechanism of regulating the phenotype of breast cancer cells. Here, we show that also in canine mammary cancer cells, BET inhibition by (+)-JQ1 had no effect on the expression of MYC in cells cultured both in 2D and 3D *in vitro* models. Furthermore, when comparing expression levels of BET proteins, we found that *BRD2* showed higher expression levels compared to *BRD3* and *BRD4* in the three cell lines, suggesting that BRD2 could be a major target of (+)-JQ1 in canine mammary cancers. In fact, a recent study has shown that BET proteins could have opposing roles in epithelial-mesenchymal transition (EMT) of HR and TNBC breast cancer. BRD2 positively regulated EMT, whereas BRD3 and BRD4 repressed EMT^27^. However, more detailed studies are needed to elucidate the precise role of BRD2 in breast cancer.

Non-toxic doses of (+)-JQ1 decreased self-renewal and tumorigenicity and induced G2/M cell cycle arrest in CMC cells. Specifically, transcriptomic analysis by RNA-seq showed an upregulation of G2/M cell-cycle arrest genes including *BTG2*, *CCNG2* and *EGFR* genes intimately associated with cell cycle control^46,47^. Previous studies showed G2/M cell-cycle arrest induced by upregulation of *CCNG2* and *BTG2* in human breast cancer cells^48,49^. In contrast to the present result, some studies showed that (+)-JQ1 can increase the number of cells in G1 phase and reduce the proportion in G2/M^50,51^. In addition, we found *BCL2L11* to be upregulated in (+)-JQ1-treated tumorspheres. BCL2L11, also known as BIM, is a pro-apoptotic protein that leads the Bax activation, which is responsible to regulate the mitochondrial pathway to apoptosis^52^. Similar results were demonstrated in another study with B-cell Lymphoma, showing that BET proteins can induce apoptosis regulating epigenetically BCL-2 family proteins^53^.

Several lines of evidence support a role of BET proteins in the regulation of CSCs. First, BET inhibition by (+)-JQ1 had a profound impact on global gene expression in tumorspheres. DE genes were enriched in pathways related to extracellular matrix and collagen organization, RNA and glycosaminoglycan metabolism, MET signaling and regulation of insulin-like growth factor (IGF). In particular, we observed that BET inhibition by (+)-JQ1 downregulated several genes of the IGF pathway including *CHRDL1, GPC3, SPP2, MXRA8, GAS6, BMP4, PAPPA*, and *FAM20A*. Insulin growth factor signaling is considered a critical factor for cancer stem cell survival and maintenance of the self-renewal phenotype^32,54–56^. In particular, GPC3 has been suggested as a promising target for immunotherapy^57,58^ and BMP4 is a well-known factor necessary for maintenance of self-renewal, EMT and CSC phenotypes. Additionally, (+)-JQ1 decreased the expression of ZEB2 transcription factor under 2D and 3D conditions. Recently, our group showed that CF41.Mg cells exhibit higher expression of ZEB2 in comparison with less tumorigenic CMC cells, suggesting a key role for ZEB2 in tumorigenicity and self-renewal of CMC cells^11^. The results described in this work, open the possibility to epigenetically inhibit ZEB2 expression by targeting BET proteins in cancer cells.

Targeting self-renewal pathways is an efficient strategy to reach more tumorigenic cells, such as CSCs^6^. Nevertheless, few studies have demonstrated a direct effect of BET proteins on self-renewal-associated pathways. In human breast cancer, (+)-JQ1 reduced the number of TNBC spheroids. However, the study focused on the effect of (+)-JQ1 on TNBC response induced by hypoxia^26^. Venkataraman *et al*. have demonstrated that BET inhibition by (+)-JQ1 suppressed stem cell-associated signaling in medulloblastoma cells and inhibited medulloblastoma tumor self-renewal^28^. Also, BET inhibition by (+)-JQ1 has been suggested to repress cell growth and modulated WNT signaling from mesenchymal stem cells without inducing apoptosis^59^.

At present, only two studies examine the role of BET proteins in canine cancer. In the first study, BRD4 was considered a novel marker and promising target in advanced mast cell neoplasms both in human and dogs^60^. The other study presented the BET inhibitor CPI-0610 with good results and acceptable toxicity, however, dogs were used only as experimental models to preclinical trials, not as a model to describe how such proteins work on tumor progression^29^. Therefore, the present study contributes to our understanding of the role of BET proteins in the biology of CMCs, suggesting BET proteins as potential therapeutic target in CMCs.

In conclusion, our findings support a role for BET inhibitors in restraining self-renewal and tumorigenicity of CMC cells by altering the expression of known cancer-associated genes. This corroborates analogous studies in human cancer and highlights BET proteins as targets for the development of innovative cancer therapies for human and dogs. In addition, the results suggest that the mechanisms responsible for obtaining these phenotypes are similar in canine and human mammary cancer, underlining the validity of canine models for comparative and translational studies.

## Methods

### Cell lines

Three cell lines were used in this experiment: M5 and M25 cells were isolated and characterized in our laboratory, as previously described^61^, and the CF41.Mg cell line, kindly provided by Dr. Debora A. P. C. Zuccari (Faculdade de Medicina de São José do Rio Preto, São José do Rio Preto, São Paulo, Brazil). All CMC cells were maintained in 75 cm^2^ flasks at 37 °C and 5% CO_2_ with Dulbecco’s Modified Eagle Medium: Nutrient Mixture F-12 (DMEM-F12) supplemented with 10% fetal bovine serum and 1% antibiotic/antimycotic. Passaging was performed when cells were 85% confluent. Culture evolution was evaluated daily by optical microscopy (Axio Vert A1, Zeiss, Germany). All reagents used for cell culture were purchased from Thermo Fisher Scientific, USA. The molecular validation of CMC cell lines is described in the Supplementary Information.

### Epigenetic probes cytotoxic assay

Epigenetic probes (Cayman Chemical, USA) were dissolved in DMSO to a concentration of 20 mM (Table 1). The CF41.Mg cells were seeded at 2000/well in 96 well plates (Corning, USA) containing 100 µl of supplemented media as described. After 24 h, media was replaced by new culture media containing different concentrations of epigenetic probes, ranging from 10 µM to 0.00064 µM. Epigenetic probes were added in six replicates per concentration. After 72 h, 10 µl of 3-(4.5-dimethylthiazol-2-yl)-2.5-diphenyl tetrazolium bromide (MTT - 5 mg/mL) was added to each well and formazan crystals were produced over a 2 h incubation period. One hundred µl of DMSO were added to dissolve crystals. Optical density at 540 nm was measured in a Fluorstar Optima (BMG Labtech, Germany). The concentration of compounds resulting in IC_50_ was calculated for each cell line using nonlinear regression test performed in GraphPad Prism (version 6.00 for Windows, GraphPad Software, USA).

### Tumorspheres formation assay

Single cells were seeded into an ultra-low attachment surface 24-well plate (Corning) at a density of 8 × 10^2^ cells suspended in 0.5 mL of serum-free DMEM-F12 supplemented with 1x B27 (Thermo Fisher Scientific), 20 ng/ml of EGF (PrepoTech, USA), 10 ng/ml of FGF (PrepoTech), 5 µg/ml of bovine insulin (Sigma Aldrich, USA), 4 µg/ml of heparin and 1% antibiotic/antimycotic. Tumorspheres number were evaluated 4 days after seeding. To generate secondary tumorspheres, primary tumorspheres were dissociated with trypsin (TrypLE Express Enzyme, Thermo Fisher Scientific). Single cells in suspension were seeded in the same density and evaluated 4 days after seeding. Pictures were taken with optical microscopy (Axio Vert A1, Zeiss).

### Soft agar assay

Single cells were mixed in 0.3% agar (in DMEM-F12 supplemented with 10% FBS and 1% antibiotic/antimycotic) and plated at 1 × 10^4^ onto 6-well plates containing a solidified bottom agar layer (0.6% agar in the same growth medium). Cells were maintained at 37 °C and 5% CO_2_ for 14 days. Colonies were photographed in 10 pattern fields, counted and measured using ZEISS ZEN 2 Microscope Software (ZEISS).

### Real-time PCR (qPCR)

Cells were treated with DMSO or 100 nM (+)-JQ1. After 72 h, total RNA was extracted using Trizol® following the manufacturer’s instructions. RNA samples were quantified and the 260/280 and 260/230 ratio (Supplementary Table S3) was assessed by NanoDrop 2000^TM^ (Thermo Fisher Scientific). cDNA was synthesized from 1 mg of total RNA using the High Capacity cDNA Reverse Transcription kit. Gene expression analyses were performed by real-time PCR using a StepOne System (Thermo Fisher Scientific). Specific primers were designed with Primer-BLAST^62^ and dimers and hairpins were verified using AutoDimer software^63^. Primers were also analyzed by in silico PCR (https://genome.ucsc.edu/cgi-bin/hgPcr) to confirm specificity. Primer sequences are reported in Supplementary Table S4. PCR reactions were carried out using Fast SYBR Green Master Mix in a final volume of 10 µl. Conditions for quantitative PCR were as follows: 95 °C for 20 s; 40 cycles at 95 °C for 3 s for denaturation, 60 °C for 30 s for anneal/extend; melt curve analysis was performed at 95 °C for 15 s and 60 °C for 60 s. The housekeeping gene used was the 18 s ribosomal RNA and the analysis of relative gene expression data was performed according to the ΔΔCt method^64^. Experiments were performed twice and in biological triplicates. All the reagents were purchased from Thermo Fisher Scientific.

### *In silico* analysis for docking (+)-JQ1 into the canine BET proteins structure

The amino acid sequences (FASTA) of human/canine BRD2 (NP_001106653.1/NP_001041552.1), BRD3 (NP_031397.1/XP_858014.1) and BRD4 (NP_490597.1/XP_013977515.1) were compared by Protein Blast^65^. Computational analysis was performed using the crystal structure of the canine BET proteins co-crystallized with (+)-JQ1 (pdb 3MXF)^18^. Receptor target and docking ligands were prepared using Chimera^66^. The molecular surface of the target was generated based on the algorithm development^67^. Sphere generation was performed using the sphgen algorithm; the spheres were distributed with dock6 and selected using “spheres_selector”. Grid generation was achieved using Grid, which is distributed as an accessory to DOCK^68^. Flexible Dock was used to verify interactions between the target BET protein and (+)-JQ1^69^. Results obtained by docking were visualized and analyzed on Chimera version 1.4.1 (build 30365).

### Cell cycle assay

The CF41.Mg cells were treated with DMSO (control) or 100 nM (+)-JQ1 for 72 h. Cells were harvested and 1 × 10^6^ cells were resuspended in cold PBS and fixed with absolute ethanol for 30 minutes. Cells were treated with 0.1% of Triton X-100 (Sigma Aldrich, USA), 20 µg/ml of propidium iodide (PI) (Thermo Fisher Scientific) and 200 µg/ml of RNase A (Thermo Fisher Scientific) for 30 minutes covered from light. Flow Cytometric Analysis was performed using S3e^TM^ Cell Sorter (Bio-Rad, USA). The data were analyzed using FCS Express 6 Flow Cytometry Software (De Novo Software, USA).

### Cell death assay

To discriminate which type of cell death (+)-JQ1 induces in CF41.Mg cells (apoptosis versus oncosis) acridine orange assay was performed which is based on the arrangement of chromatin to differentiate apoptotic, oncotic and live cells. Live cells have normal nuclei staining which presents green chromatin with organized structures. Apoptotic cells contain condensed or fragmented chromatin (green or orange) and oncotic cells have similar normal nuclei staining as live cells except the chromatin is orange instead of green^70^. The CF41. Mg cells were seeded in 6-well plates and after 24 h, cells were treated with DMSO or (+)-JQ1 at a final concentration of 4 µM, 1 µM, 300 nM, 150 nM and 100 nM for 72 h. A dye mix containing 100 µg/ml of acridine orange and 100 µg/ml of ethidium bromide was added to cells and observed for fluorescence emission using ZEISS—Axio Vert A1 with a camera Axio Can 503 attached using a 520 nm and 620 nm wavelength filter for green and red colors, respectively (ZEISS). Analyzes were performed in triplicate, counting a minimum of 100 total cells each.

### RNAseq data generation

Tumorspheres treated with 100 nM (+)-JQ1 or 100 nM (−)-JQ1 were collected after 4 days of culture and the RNA was extracted using RNeasy Mini Kit (QIAGEN, UK). The RNA quality and quantity were assessed using automated capillary gel electrophoresis on a Bioanalyzer 2100 with RNA 6000 Nano Labchips according to the manufacturer’s instructions (Agilent Technologies, Ireland). Only samples that presented an RNA integrity number (RIN) higher than 8.0 were considered to the sequencing (Supplementary Table S5). RNA libraries were constructed using the TruSeq™ Stranded mRNA LT Sample Prep Protocol and sequenced on Illumina HiSeq. 2500 equipment in a HiSeq Flow Cell v4 using HiSeq SBS Kit v4 (2 × 100 pb).

### Alignment and differential expression

Sequencing quality was evaluated using the software FastQC (http://www.bioinformatics.babraham.ac.uk/projects/fastqc) and no additional filter was performed. Sequence alignment against the canine reference genome (CanFam3.1) was performed using STAR^71^, according to the standard parameters and including the annotation file (Ensembl release 89). Secondary alignments, duplicated reads and reads failing vendor quality checks were removed using Samtools^72^. Alignment quality was confirmed using Qualimap^73^. Gene expression was estimated by read counts using HTseq^74^ and normalized as counts per million reads (CPM). Only genes presenting at least 1 CPM in at least 6 samples were kept for differential expression (DE) analysis. DE was performed using EdgeR package^75^ on R environment, based on negative binomial distribution. Benjamini-Hochberg procedure was used to control the false discovery rate (FDR) and transcripts presenting FDR ≤ 0.01 and log-fold change (LogFC) > 1 were considered differential expressed (DE). Functional enrichment analysis of DE genes was performed using STRING^76,77^.

### Statistical Analysis

The IC_50_ was calculated using nonlinear regression test. Gene expression, colonies and tumorsphere formation were analyzed by one-way ANOVA with post hoc Tukey. Unpaired T-test was used for gene expression analysis of non-treated and (+)-JQ1-treated cells. For functional enrichment analyses, P-value was adjusted for multiple tests, and Benjamini and Hochberg method was used to test multiple categories in a group of functional gene sets. Significant differences were considered when p < 0.05.

## Supplementary information

Supplementary data

Supplementary Table S2

## Data Availability

All data generated or analyzed during this study are included in this published article (and its Supplementary Information files). The datasets generated during and/or analyzed during the current study are available from the corresponding author on reasonable request.
