# Supplementary material for: An epigenetic screening determines BET proteins as targets to suppress self-renewal and tumorigenicity in canine mammary cancer cells
Source: Sci Rep. 2019 Nov 22;9:17363. doi: 10.1038/s41598-019-53915-7 (PMC6874531; doi:10.1038/s41598-019-53915-7)
Supplement: Supplementary file 1 — Supplementary data [file 41598_2019_53915_MOESM1_ESM.docx]

**An epigenetic screening determines BET proteins as targets to suppress self-renewal and tumorigenicity in canine mammary cancer cells**

Pedro L. P. Xavier^1^, Yonara G. Cordeiro^1^, Pâmela A. Alexandre^1,2^, Pedro R. L. Pires^1^, Bruno H. Saranholi^3^, Edson R. Silva^4^, Susanne Müller^5^, Heidge Fukumasu^1*^

^1^ Laboratory of Comparative and Translational Oncology (LOCT), Department of Veterinary Medicine, Faculty of Animal Science and Food Engineering, University of Sao Paulo, Pirassununga, Brazil

^2^ Present address: CSIRO Agriculture and Food, Commonwealth Scientific and Industrial Research Organisation, Brisbane, Australia

^3^ Departament of Genetics and Evolution, Federal University of São Carlos, São Carlos, Brazil

^4^ Department of Veterinary Medicine, Faculty of Animal Science and Food Engineering, University of Sao Paulo, Pirassununga, Brazil

^5^ Structural Genomics Consortium, Buchmann Institute for Molecular Life Sciences, Johann Wolfgang Goethe University, Frankfurt am Main, Germany

***Correspondence to:** Heidge Fukumasu, **email:** [fukumasu@usp.br](mailto:fukumasu@usp.br)

**Supplementary data**

**Molecular validation of CMC cells**

In order to confirm the origin of cell lines, we amplified a fragment from cytochrome oxidase I (COI) and from 16S region using the primers described by Folmer (1994) and Palumbi et al. (1991) ^1,2^. For both regions, the PCR reactions were performed at a final volume of 12 µL, containing: 0.2 mM of dNTPs, buffer tris-KCl 1X (Tris–HCl 20 mM pH 8.4 and KCl 50 mM), 2.5 mM of MgCl_2_ (Invitrogen), 0.8 mM of each primer pair, 1 unit of Taq DNA Polymerase Platinum (Invitrogen), 50 ng of DNA template and ultrapure water (q.s.). The PCRs were conducted in Veriti 96 Well Thermal Cycler (Applied Biosystems, Foster City, CA, USA) with an initial denaturation step at 94°C for 1 min; 35 cycles at 95°C for 30 s, the annealing temperature for each primer (50°C for COI and 57°C for 16S) for 1min and 72°C for 1 min; and a final extension step at 72°C for over 10 min. DNA from leukocytes of a known dog (*Canis lupus familiaris*) was used as positive control for PCRs. We also included a reference DNA from human and murine for the further cell lineage confirmation analysis. The amplification products were verified in electrophoresis on agarose gel at 1%.

The PCR products were purified using ExoSAP-IT (Affymetrix) and sequenced in an ABI3730XL sequencer (Applied Biosystems). The obtained sequences were analyzed and aligned with a reference sequence of a dog using the Geneious v.7.1.7 software ^3^. A sequence from *Felis catus* was used as outgroup (GenBank accession number FJ958339.1 – COI region, and GenBank accession number DQ983942 – 16S). The human and murine sequences obtained were also included as reference sequences. Genetic distances among the sequences were obtained using Kimura two-parameter model ^4^ and a neighbor-joining tree ^5^ was constructed using 1000 bootstrap replicates, both implemented in Geneious v.7.1.7 software ^3^ (**Supplementary figure S8 and S9**). Also, the cell lineage confirmation was obtained comparing the obtained sequences with the sequences available in GenBank ^6^ using the Blast tool ^7^.

**Supplementary references**

1. Folmer, O., Black, M., Hoeh, W., Lutz, R. & Vrijenhoek, R. DNA primers for amplification of mitochondrial cytochrome c oxidase subunit I from diverse metazoan invertebrates. *Mol. Mar. Biol. Biotechnol.* **3**, 294–299 (1994).

2. Palumbi, S., Romano, S., Mcmillan, W. O. & Grabowski, G. the Simple Guide To Pcr. **96822**, 1–45 (2002).

3. Kearse, M. *et al.* Geneious Basic: An integrated and extendable desktop software platform for the organization and analysis of sequence data. *Bioinformatics* **28**, 1647–1649 (2012).

4. Kimura, M. Evolutionary Rates models. *J. Mol. Evol* **16**, 111–120 (1980).

5. Saitou, N. & Nei, M. The neighbor-joining method: a new method for reconstructing phylogenetic trees. *Mol. Biol. Evol.* **4**, 406–425 (1987).

6. Benson, D. A. *et al.* GenBank. *Nucleic Acids Res.* **43**, D30–D35 (2015).

7. Ye, J. *et al.* Primer-BLAST: a tool to design target-specific primers for polymerase chain reaction. *BMC Bioinformatics* **13**, 134 (2012).

**Supplementary Tables**

**Supplementary Table S1.** Alignment rates of control (C) and (+)-JQ1-treated tumorspheres (J).

| **Samples** | **Number of input reads** | **Average input read length** | **Uniquely mapped reads number** | **% Uniquely mapped reads** | **Number of reads mapped to multiple loci** | **% of reads mapped to multiple loci** | **Number of reads mapped to too many loci** | **% of reads mapped to too many loci** | **% of reads unmapped: too short** |
| --- | --- | --- | --- | --- | --- | --- | --- | --- | --- |
| C1 | 21134309 | 198 | 19250789 | 91.09 | 1127917 | 5.34 | 54134 | 0.26 | 3.25 |
| C2 | 18900257 | 199 | 17242791 | 91.23 | 1001814 | 5.30 | 47513 | 0.25 | 3.16 |
| C3 | 19065644 | 199 | 17392134 | 91.22 | 1039665 | 5.45 | 47738 | 0.25 | 3.01 |
| J1 | 18531549 | 199 | 16564896 | 89.39 | 1077535 | 5.81 | 43377 | 0.23 | 4.51 |
| J2 | 21907957 | 198 | 19824356 | 90.49 | 1208771 | 5.52 | 57637 | 0.26 | 3.67 |
| J3 | 19379659 | 198 | 17441950 | 90 | 1131391 | 5.84 | 48753 | 0.25 | 3.85 |

**Supplementary Table S3**: Concentration, 260/280 nm and 260/230 nm ratio of the RNA samples.

| Samples | RNA Concentration (ng/µl) | 260/280 | 260/230 |
| --- | --- | --- | --- |
| C1 | 434.3 | 1.95 | 2.2 |
| C2 | 537.9 | 1.94 | 2.3 |
| C3 | 405 | 1.93 | 2.3 |
| J1 | 275.5 | 1.98 | 1.9 |
| J2 | 269.9 | 1.95 | 2.2 |
| J3 | 233.4 | 1.95 | 2.1 |

**Supplementary Table S4.** Gene-specific primer sequences used for real time quantitative PCR.

| **Primers** | **Sequence** |
| --- | --- |
| 18S (NR_003278.3) | F: 5’-CCTGCGGCTTAATTTGACTC-3’  R: 5’-CTGTCAATCCTGTCCGTGTC-3’ |
| BRD2 ([NM_001048087.1](https://www.ncbi.nlm.nih.gov/entrez/viewer.fcgi?db=nucleotide&id=114326382)) | F:5’-GGAGTTTGCTGCTGATGTGC-3’ |
|  | R:5’-CCCTGGTTCCAGTGGTTCAT-3’ |
| BRD3 ([XM_005625157.3](https://www.ncbi.nlm.nih.gov/entrez/viewer.fcgi?db=nucleotide&id=1239920669)) | F:5’-GCAATCAAGCTGAACCTGCC-3’ |
|  | R:5’-GTCCTGCATACATTCGCTCG-3’ |
| BRD4 ([XM_014122040.2](https://www.ncbi.nlm.nih.gov/entrez/viewer.fcgi?db=nucleotide&id=1239951039)) | F:5’-GCCACCGTTCCAGAGCC-3’ |
|  | R:5’-TCCTCCTTCACCACCACCA-3’ |
| CDH1 (NM_001287125.1) | F: 5’-AGGCCCATTTCCTAAAAACC-3’  R: 5’-TTGGCCAGTGATGCTGTAGA-3’ |
| C-Myc ([NM_001003246.2](https://www.ncbi.nlm.nih.gov/entrez/viewer.fcgi?db=nucleotide&id=153070852)) | F:5’-TTTGGACGCTGGATCTCCTC-3’ |
|  | R:5’-AAGCTGACGTTGAGAGGCAT-3’ |
| SLUG (NM_001097981.1) | F: 5’-CGTTTTCCAGACCCTGGTTA-3’  R: 5’-GCAGTGAGGGCAAGAAAAAG-3’ |
| SOX2 ([XM_005639752.3](https://www.ncbi.nlm.nih.gov/entrez/viewer.fcgi?db=nucleotide&id=1239978791)) | F: 5’-GTCCCAGCACTACCAGAGCG-3’ |
|  | R:5’-CTTACTCTCCTCCCATTTCCCTCG-3’ |
| STAT3 (XM_005624457.2) | F: 5’-GCCAATGCTAGAGGAGAGGA-3’  R: 5’-AACTGGACGCCAGTCTTGAT-3’ |
| ZEB1 (XM_005617037.2) | F: 5’-TTGCTCCCTGTGCAGTTACA-3’  R: 5’-AGCTTTCCCACATTCAGTGC-3’ |
| ZEB2 (XM_005631964.2) | F: 5’-ACCCAGGACTGGATCAGATG-3’  R: 5’-GCTCCATCAAGCAATTCTCC-3’ |

**Supplementary Table S5.** RNA concentration and RNA integrity number (RIN) of control tumorspheres (C) and treated with 100 nM (+)-JQ1 (J).

| Samples | Concentration (ng/µl) | RIN |
| --- | --- | --- |
| C1 | 326 | 10 |
| C2 | 346 | 10 |
| C3 | 426 | 10 |
| J1 | 117 | 10 |
| J2 | 152 | 10 |
| J3 | 157 | 9.7 |

**Supplementary Figures**


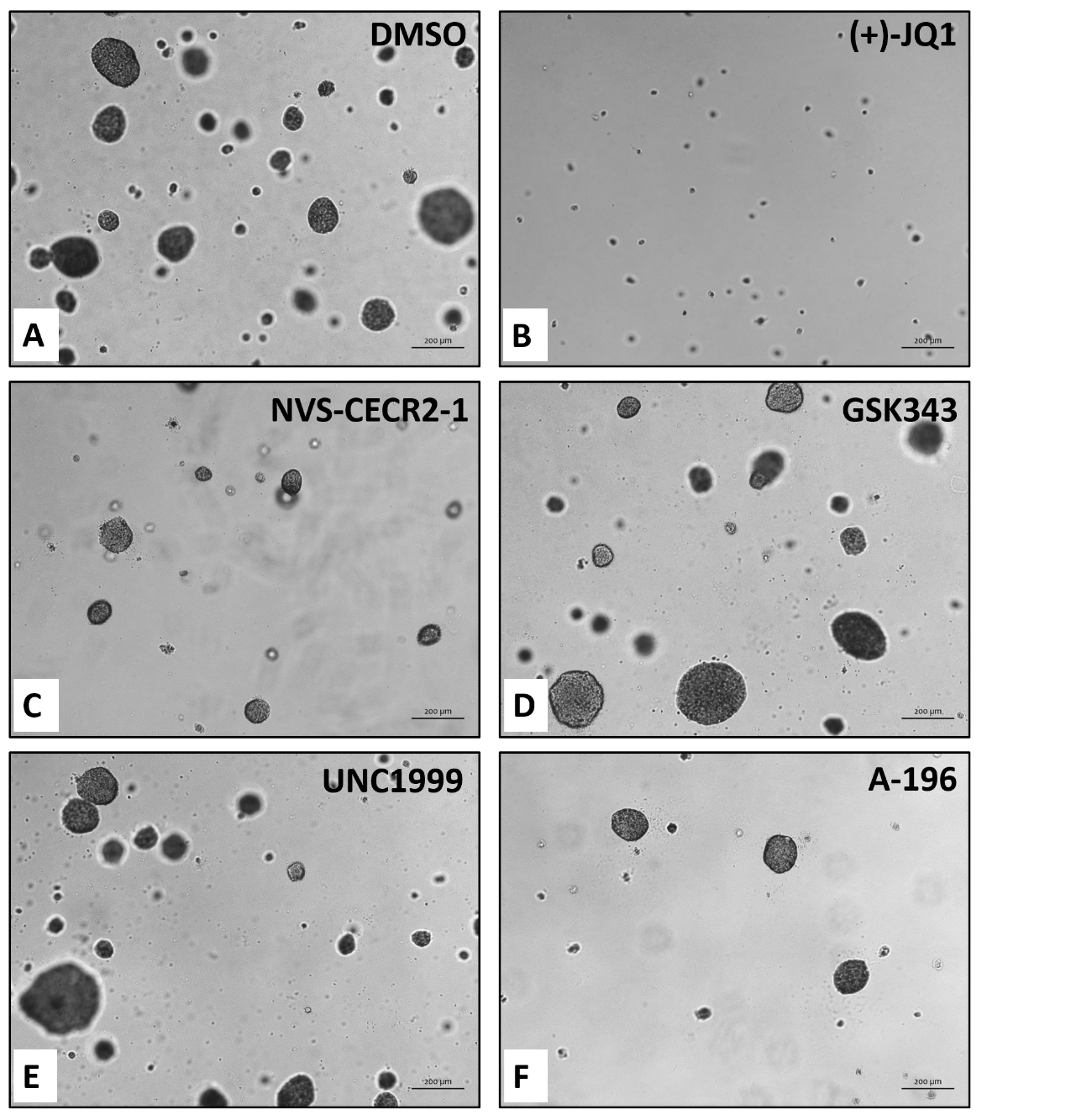


**Supplementary figure S1.** 27 epigenetic inhibitors were tested for colonies formation in soft agar assay. Only (+)-JQ1 (**B**), NVS-CECR2-1 (**C**), GSK343 (**D**), UNC1999 (**E**) and A-196 (**F**) decreased the number of colonies in comparison to the control (DMSO) (**A**). Images were obtained in a 5x objective.

**
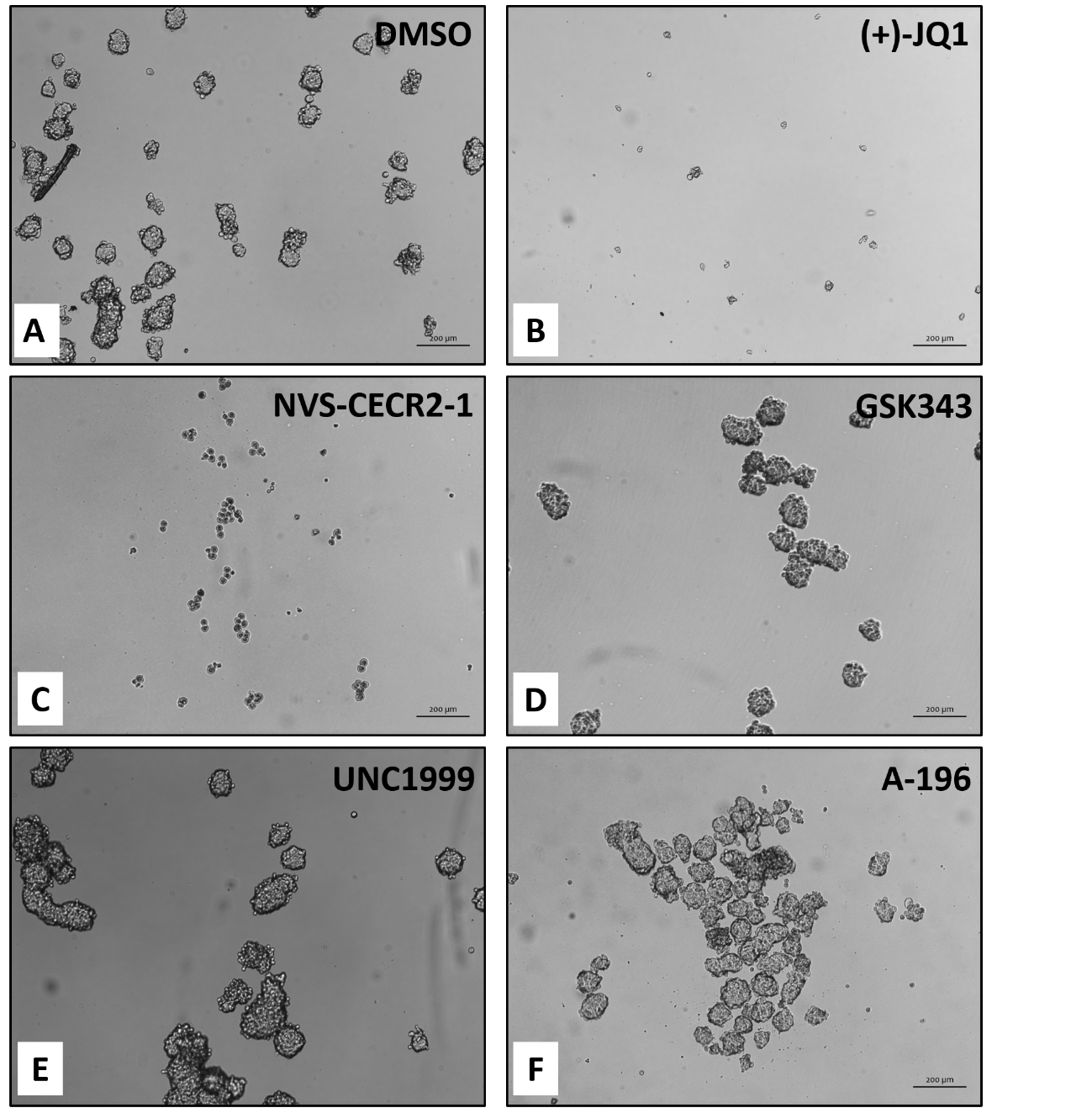
**

**Supplementary figure S2.** Only (+)-JQ1 (**B**) and NVS-CECR2-1 (**C**) were able to inhibit the tumorsphere formation in low-adherent plates. No difference was observed to tumorspheres treated with GSK343 (**D**), UNC1999 (**E**), and A-196 (**F**) in comparison to the control (DMSO) (**A**). Images were obtained in a 5x objective.

**
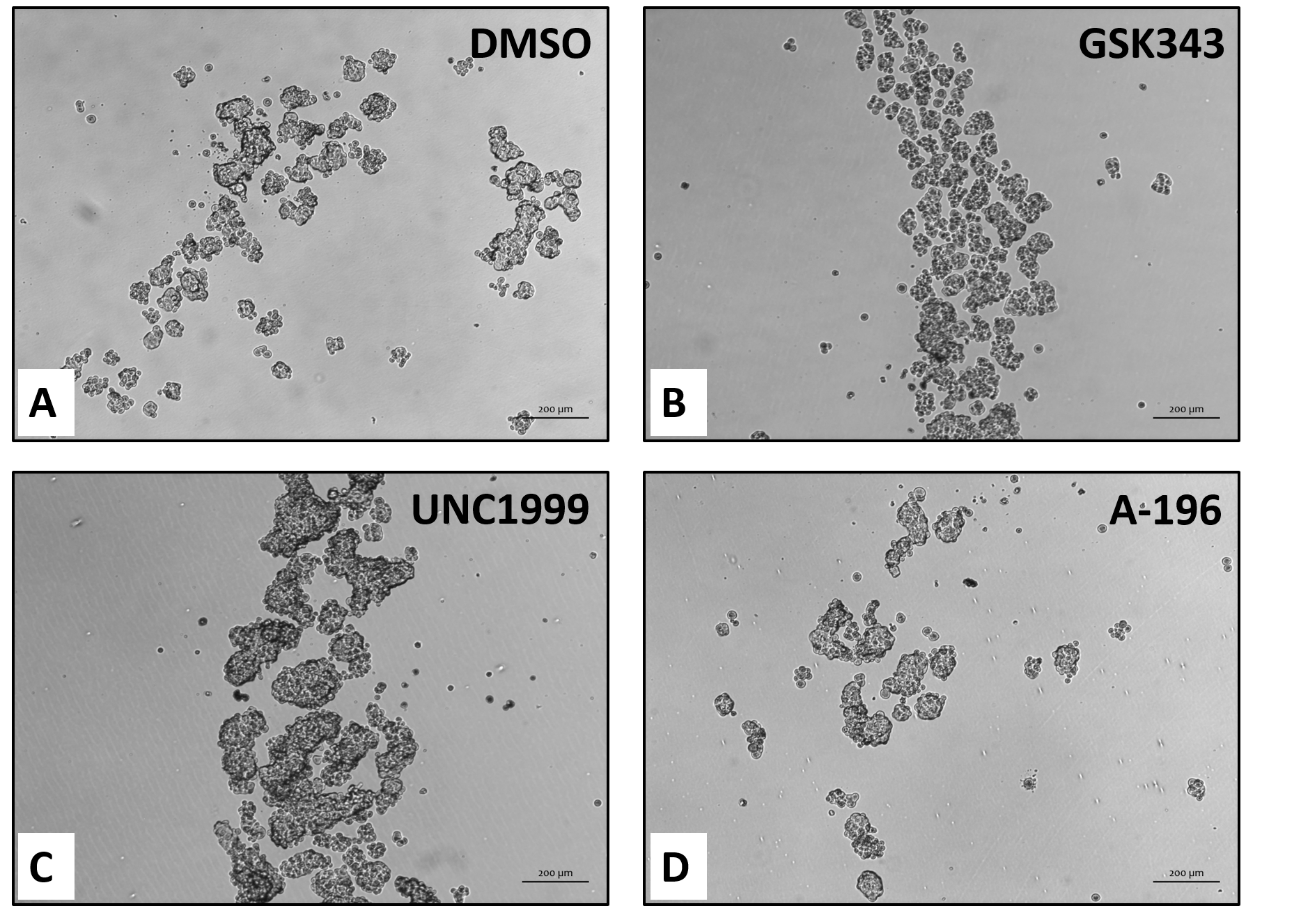
**

**Supplementary figure S3.** GSK343 (**B**), UNC1999 (**C**) and A-196 (**D**) were also unable to inhibit the formation of secondary tumorspheres in comparison to the control (**A**). Images were obtained in a 5x objective.

**
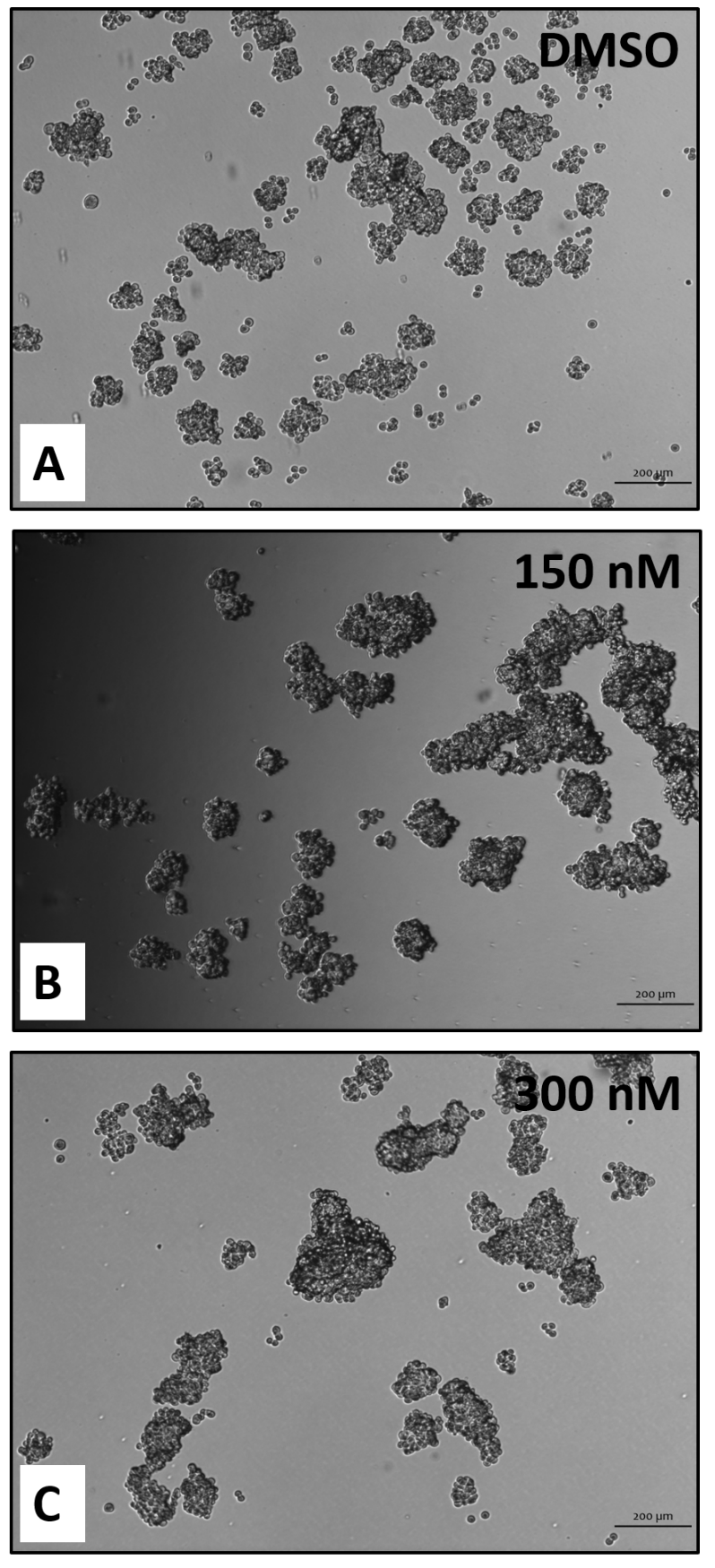
**

**Supplementary figure S4.** Lower doses of 150 nM (**B**) and 300 nM (**C**) NVS-CECR2-1 did not show the same effect on CF41.Mg tumorspheres in comparison to the control (**A**). Images were obtained in a 5x objective.

**
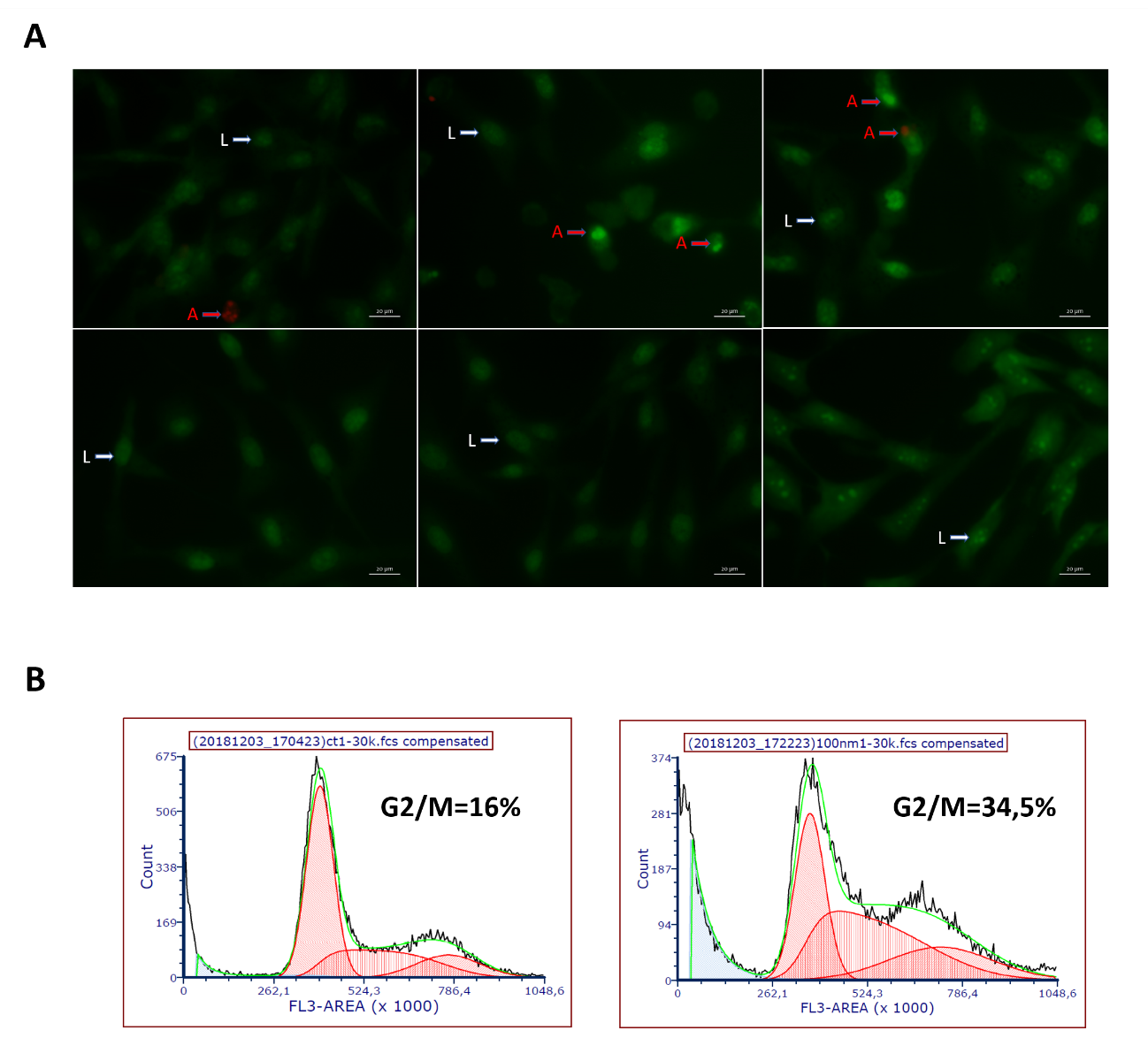
**

**Supplementary figure S5. (A)** Four µM or 1 µM, respectively of (+)-JQ1 induced apoptosis in CF41.Mg cells. On the other hand, 300 nM, 150 nM and 100 nM of (+)-JQ1 showed no difference in comparison to the control (L= Live cells; A= Apoptotic cells). (**B)** Flow cytometry histograms for CF41.Mg cells show increase G2/M cell cycle arrest in (+)-JQ1 treated cells compared to the control.

**
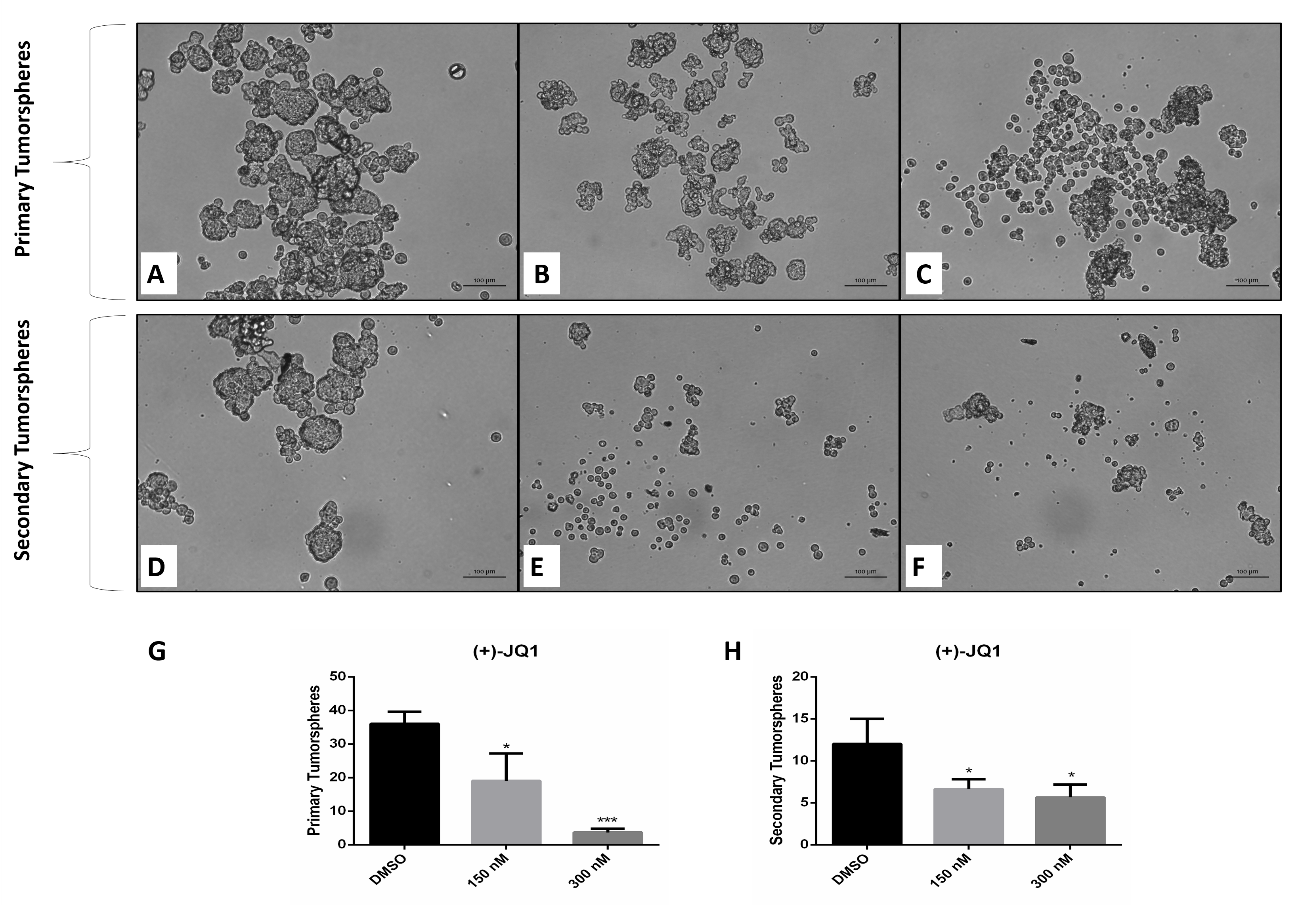
**

**Supplementary figure S6.** (+)-JQ1 at concentrations of 150 nM (**B, E**) and 300 nM (**C, F**) reduced the number of primary and secondary tumorspheres of M5 cells in comparison to the control (DMSO) (**A, D**). Images were obtained in a 5x objective. (*p< 0.05; *** p <0.001 - One way ANOVA followed by Tukey's multiple comparison test).

**
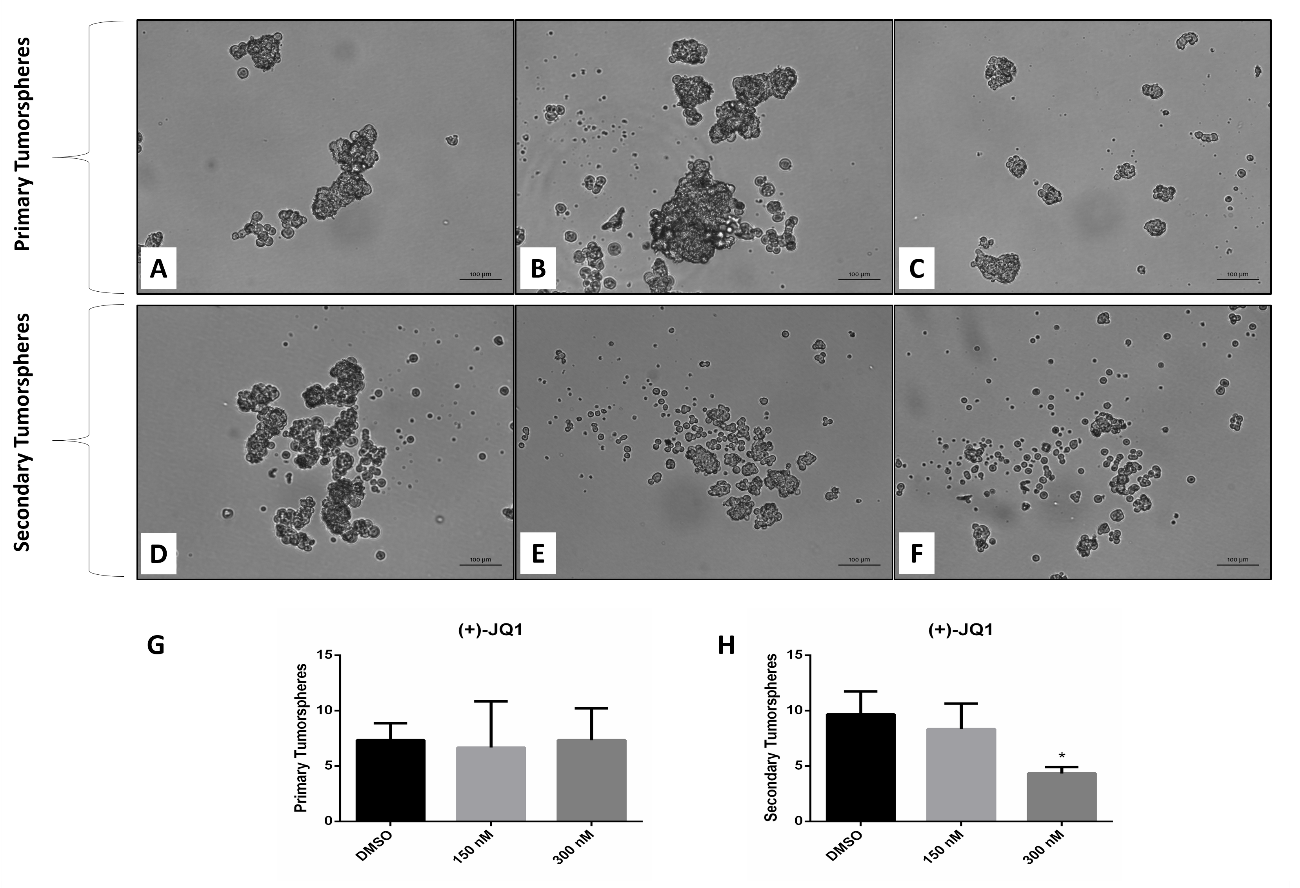
**

**Supplementary figure S7.** In M25 cells, only the number of secondary tumorspheres was reduced, when treated with 300 nM (+)-JQ1 (**F**). Primary tumorspheres number was not affected by low concentrations of (+)-JQ1 (**B, C**) in comparison to the control (DMSO) (**A**). The concentration of 150 nM of (+)-JQ1 was not able to decrease the number of secondary tumorspheres (**E**) in comparison to the control (DMSO) (**D**). Images were obtained in a 5x objective. (*p< 0.05 - One way ANOVA followed by Tukey's multiple comparison test).


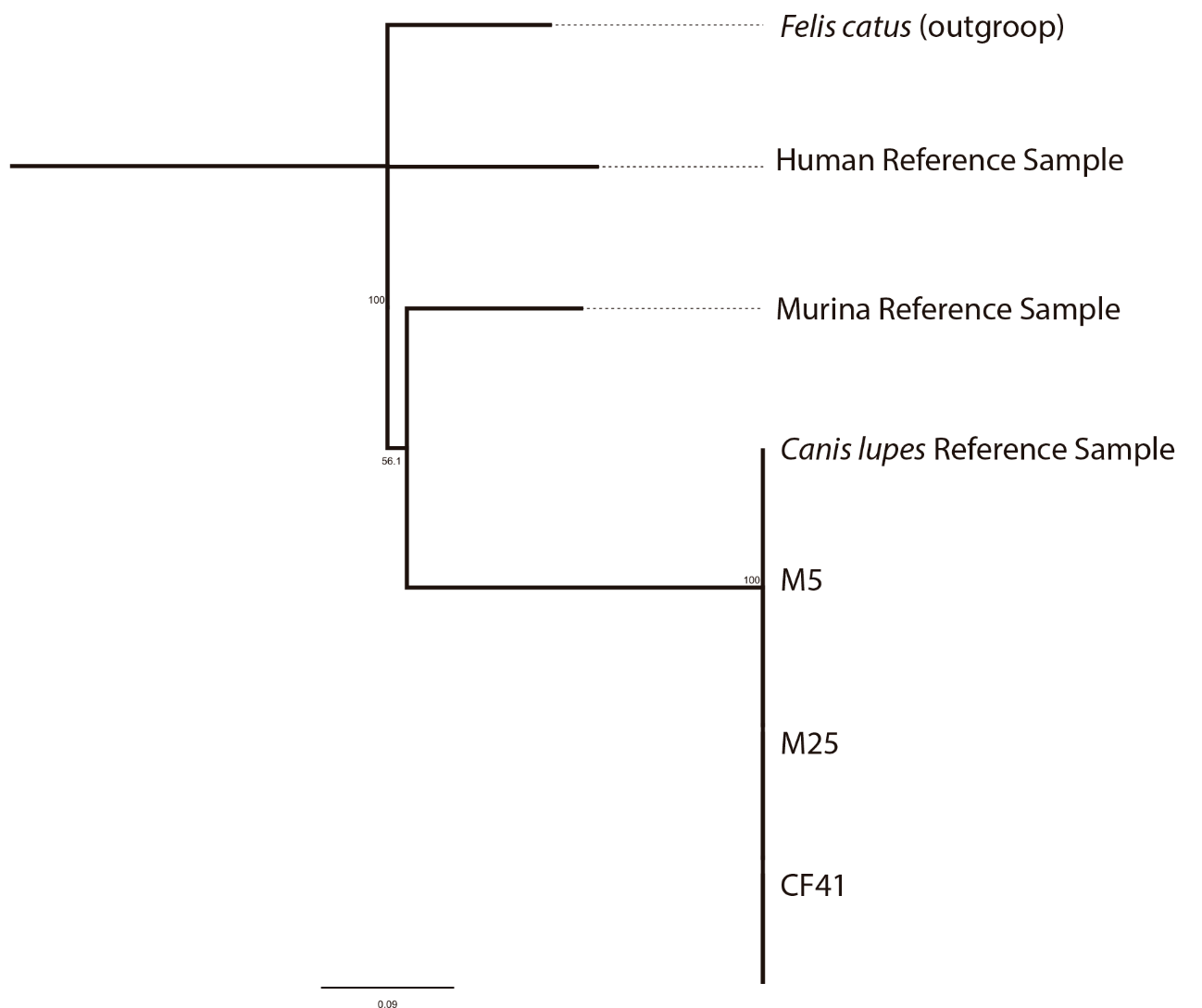


**Supplementary figure S8.** COI Neighbor Joining tree of cell lineage confirmation. M5, M25 and CF41 represents cell lineages evaluated. The *Canis Lupus* reference sample DNA was obtained from leukocytes of a known dog (*Canis lupus familiaris*). The human and murine reference DNA samples were obtained from H460 human lung cancer cell line and E9 murine cell, respectively. *Felis catus* (outgroup) sequence obtained from GenBank (accession number J958339.1).


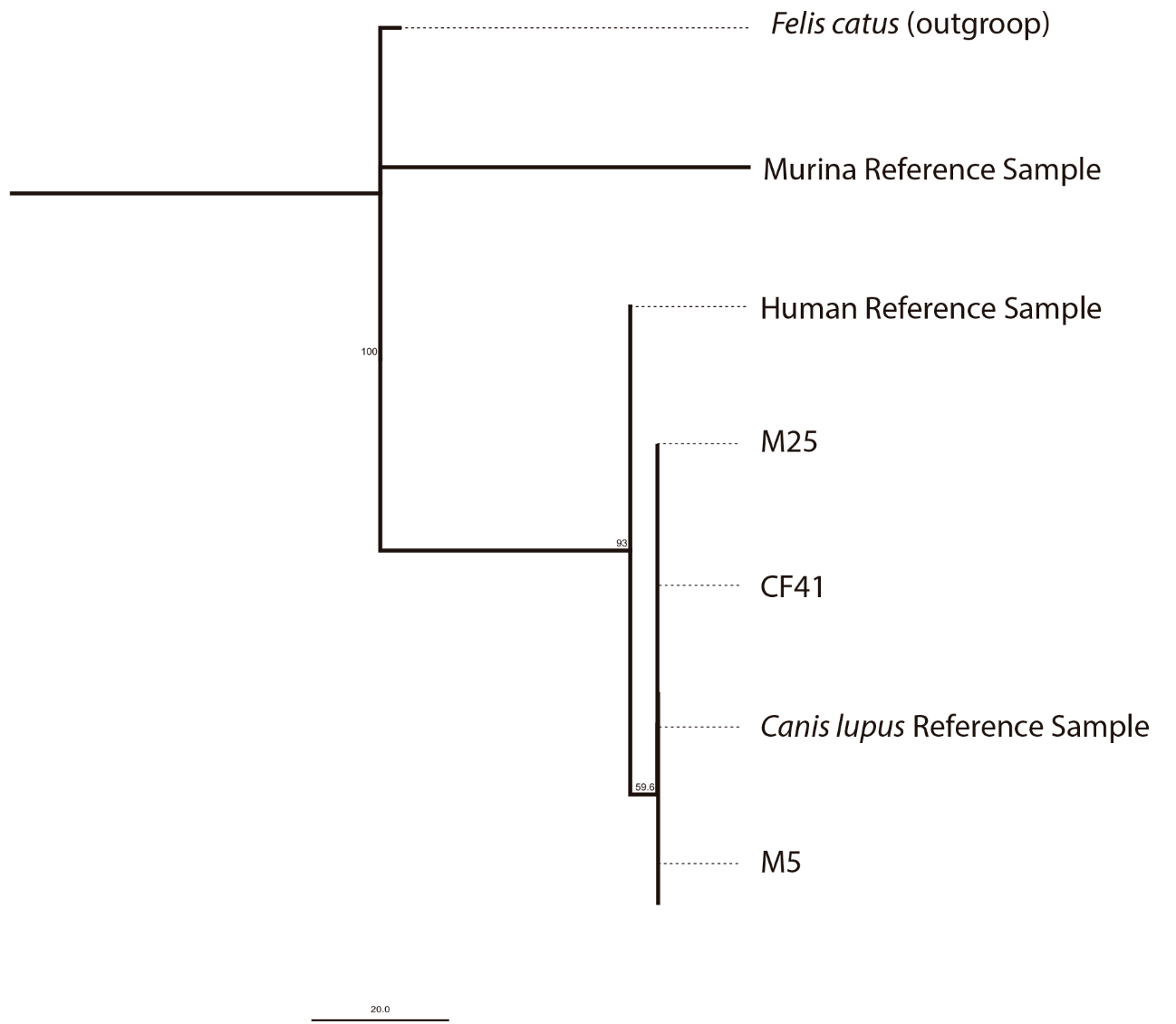


**Supplementary figure S9.** 16S Neighbor Joining tree of cell lineage confirmation. M5, M25 and CF41 represents cell lineages evaluated. The *Canis Lupus* reference sample DNA was obtained from leukocytes of a known dog (*Canis lupus familiaris*). The human and murine reference DNA samples were obtained from H460 human lung cancer cell line and E9 murine cell, respectively. *Felis catus* (outgroup) sequence obtained from GenBank (accession number DQ983942).
